# Apigenin Inhibits the Growth of Esophageal Squamous Cell Carcinoma (ESCC) Cells by Harnessing the Expression of MicroRNAs

**DOI:** 10.3390/biom16030366

**Published:** 2026-02-28

**Authors:** Nouman Amjad, Muhammad Majid, Zhaojian Sun, Rajesh Basnet, Kashaf Rasool, Linping Wu, Zhiyuan Li

**Affiliations:** 1 CAS Key Laboratory of Regenerative Biology, Guangdong Provincial Key Laboratory of Stem Cell and Regenerative Medicine, Guangzhou Institutes of Biomedicine and Health, Chinese Academy of Sciences, Guangzhou 510530, China; nouman@gibh.ac.cn (N.A.); muhammad@gibh.ac.cn (M.M.); sun_zhaojian@gibh.ac.cn (Z.S.); rajesh@gibh.ac.cn (R.B.); wu_linping@gibh.ac.cn (L.W.); 2 Department of Pathology, Faculty of Veterinary and Animal Sciences (FV&AS), The Islamia University of Bahawalpur, Bahawalpur 63100, Pakistan; 3 Atta-ur-Rahman School of Applied Biosciences (ASAB), National University of Science and Technology (NUST), Islamabad 44000, Pakistan; krasool.phdabs22asab@student.nust.edu.pk; 4 Department of Anatomy and Neurobiology, Xiangya School of Medicine, Central South University, Changsha 410013, China

**Keywords:** apigenin, ESCC, miRNAs, transcriptome sequencing, pathway analysis

## Abstract

Apigenin, a naturally occurring flavonoid with low toxicity, exhibits anticancer activity, yet its effects on microRNAs (miRNAs) and downstream gene networks in esophageal squamous cell carcinoma (ESCC) remain unclear. Here, we evaluated apigenin’s antitumor effects in TE-1 and Eca-109 cells, assessing proliferation, apoptosis, colony formation, and invasion. Differentially expressed miRNAs were identified via small RNA sequencing, and candidate target genes were predicted, annotated using GO and KEGG analyses, and validated by qRT-PCR, revealing miRNA-mediated regulatory mechanisms underlying apigenin’s inhibitory effects in ESCC. Apigenin markedly suppressed cell proliferation, clonogenic growth, wound closure, and invasive capacity, while promoting apoptosis in a dose-dependent manner. In TE-1 cells, apigenin upregulated hsa-let-7c-3p, hsa-miR-374c-3p, hsa-miR-3177-3p hsa-miR-4454, and hsa-miR-4728-3p, while downregulating hsa-miR-573, hsa-miR-548az-5p, hsa-miR-33b-5p, hsa-miR-4479, and hsa-miR-3198. Correspondingly, tumor-associated target genes including *ALDH3A2*, *SEMA3F*, *MAP4K5*, and *TRIP13* were upregulated, whereas *PIK3IP1*, *AGO2*, *MMP2*, and *RALBP1* were suppressed. In Eca-109 cells, apigenin altered the expression of distinct miRNAs, including the upregulation of hsa-miR-891-5p, hsa-miR-3170, hsa-miR-4421, and hsa-miR-675-5p and the downregulation of hsa-miR-153, hsa-miR-3188, and hsa-miR-4435, thereby modulating key oncogenic targets such as *MAPK1*, *SALL4*, and *COX15*. Functional enrichment analyses indicated that apigenin-regulated genes are involved in multiple cancer-related pathways across cytoplasmic and nuclear compartments. Overall, these results suggest that apigenin suppresses ESCC progression via coordinated miRNA–mRNA regulation, highlighting its potential as a therapeutic agent.

## 1. Introduction

Esophageal squamous cell carcinoma (ESCC) continues to represent the predominant histological form of esophageal cancer, accounting for roughly 85% of cases worldwide. While GLOBOCAN 2020 reported 604,100 new cases and 544,100 deaths, updated estimates from the Global Burden of Disease 2021 study show 576,529 new cases and 538,602 deaths in 2021. These persistent and substantial numbers highlight the ongoing global impact of ESCC and the pressing need for better prevention, earlier diagnosis, and more effective treatment strategies [1,2].

Esophageal cancer is among the most lethal malignancies globally, ranking seventh in cancer-related mortality and exhibiting an unfavorable clinical prognosis. For ESCC, the overall five-year survival rate remains low at approximately 18.9% [2]. Survival outcomes in ESCC differ substantially according to disease stage, with approximately 49% for localized tumors, 28% for regional spread, and merely 5% for distant metastases. This pronounced imbalance reflects the crucial role of early detection and prompt intervention in improving prognosis and patient survival [3]. ESCC is commonly managed with surgery, radiation, and chemotherapy, but high recurrence, metastasis, and therapy resistance continue to limit treatment success [4]. Current therapeutic options for ESCC are often costly and accompanied by significant toxicity, underscoring the urgent need for alternative treatments that are both more affordable and better tolerated [5].

Apigenin is a naturally occurring flavonoid found in numerous plants such as *Scutellaria barbata*, *Lobelia chinensis*, *Oldenlandia diffusa*, and *Caulis trachelospermi*, and is increasingly recognized for its low intrinsic toxicity and wide range of biological functions, encompassing antioxidant, anti-inflammatory, antiviral, and antitumor activities [6]. Only a limited number of studies have investigated the effects of apigenin on esophageal cancer. Available evidence indicates that Apigenin has been demonstrated to markedly suppress the proliferation and induce apoptosis in esophageal cancer cells, while concurrently suppressing angiogenesis and invasion/metastasis [7]. Additionally, apigenin appears to enhance the effectiveness of conventional treatments and mitigate their toxicity, supporting its potential role as a complementary therapeutic agent in ESCC management [8].

However, the molecular mechanisms by which apigenin regulates the growth and invasion of esophageal cancer remain insufficiently understood and warrant further comprehensive investigation.

In addition, apigenin has been shown to modulate microRNA (miRNA) expression profiles, which play critical roles in regulating cellular proliferation, apoptosis, migration, and invasion in hepatocellular carcinoma and lung carcinoma [9,10]. However, the miRNA expression profile induced by apigenin in ESCC cell lines remains unknown, underscoring the need for further mechanistic investigation.

MicroRNAs (miRNAs) are short non-coding RNA species, generally 19–22 nucleotides long, that regulate gene expression primarily at the post-transcriptional level. Through interaction with 3′ untranslated regions (UTRs) of target messenger RNAs (mRNAs), miRNAs modulate protein synthesis [11]. This regulation occurs through the degradation of mRNA or the inhibition of translation, ultimately influencing protein synthesis [12]. MicroRNAs play a crucial regulatory role in maintaining the integrity of the cell cycle, governing key processes such as cellular proliferation, differentiation, and programmed cell death [13]. The miRNAs are critically involved in cancer development and progression by modulating key regulatory networks that govern tumorigenesis [14].

Through altered expression in cancer cells, specific microRNAs (miRNAs) may act as oncogenic miRNAs (oncomiRs) or function as tumor suppressors, thereby disrupting the tightly regulated gene expression networks that control fundamental cellular processes, including proliferation, apoptosis, differentiation, and metastasis [15]. Dysregulated miRNA activity contributes to tumorigenesis by downregulating tumor suppressor genes or by amplifying oncogenic signaling pathways, ultimately fostering uncontrolled cell growth, resistance to programmed cell death, and the acquisition of other hallmarks of cancer [16]. These alterations are associated with multiple cancer hallmarks, including persistent proliferative signaling, escape from growth-inhibitory controls, resistance to programmed cell death, and increased invasive and metastatic capacity. Consequently, microRNAs are being actively explored as potential biomarkers for cancer diagnosis and prognosis, as well as therapeutic targets [17]. Apigenin has been reported to exhibit anti-ESCC pharmacological effects; however, specific miRNA molecules and associated genes that apigenin targets in the prevention and treatment of ESCC remain largely unidentified.

Therefore, in the current study, we evaluated the anticancer effects of apigenin on ESCC cell lines, TE-1 and Eca-109. To gain insights into the underlying mechanisms, we performed next-generation miRNA sequencing to analyze the differential miRNA expression profiles following apigenin treatment. Through this analysis, we identified key miRNAs and their potential target genes that may be linked to the observed growth inhibition of ESCC cells. This study further investigates the miRNA–mRNA regulatory networks and the underlying molecular pathways through which apigenin may exert its anticancer effects in ESCC.

## 2. Materials and Methods

### 2.1. Reagents and Cell Lines

Apigenin (Catalog No. CFN98843) was procured from Wuhan ChemFaces Biochemical Co., Ltd., Wuhan, Hubei, China. The human ESCC cell lines TE-1 and Eca-109 were sourced from the Cell Bank of the Chinese Academy of Sciences (Shanghai, China) and subsequently maintained by our research team. Cells were maintained in RPMI-1640 medium (Gibco, Waltham, MA, USA) enriched with 1 mM L-glutamine, 10% (*v*/*v*) fetal bovine serum (FBS) (Gibco, USA). All cell cultures were maintained at 37 °C in a humidified incubator with 5% CO_2_.

### 2.2. Cell Viability and Colony Formation Assays

For cell viability assays, TE-1 and Eca-109 cells were seeded into 96-well culture plates at 5 × 10^3^ cells per well and treated with apigenin at concentrations of 3, 5, 10, 20, 40, and 80 μM, or with 0.1% Dimethyl sulfoxide (DMSO, ≥99.9%, Ameko Life Sciences, Wuhan, China) as the vehicle control. Following 24, 48, and 72 h of incubation, cell viability was evaluated using the Cell Counting Kit-8 (CCK-8; APExBIO Technology LLC, Houston, TX, USA). According to manufacturer instructions, 10% CCK-8 working solution prepared in serum-free medium was added to each well (10 μL/well), and plates were incubated for an additional 30 min at 37 °C. Absorbance was measured at 450 nm using a Countess automated cell counter (Thermo Fisher Scientific, Waltham, MA, USA). IC_50_ values were calculated with GraphPad Prism (version 8.0.1). The colony formation assay was then performed as follows:

TE-1 and Eca-109 cells were seeded into 35 mm × 10 mm Petri dishes at a density of 1000 cells per well. Cells were treated with the indicated concentrations of apigenin for 48 h, after which the medium was replaced with fresh drug-free culture medium. Colonies were allowed to grow until visible, then fixed and stained with 0.2% crystal violet (OKA, Beijing, China), imaged, and manually counted.

### 2.3. Apoptosis Analysis

TE-1 and Eca-109 cells were seeded into 6-well plates at a density of 3 × 10^5^ cells per well and treated with apigenin (5, 10, and 20 μM) or 0.1% DMSO for 48 h. Apoptosis was evaluated using the Annexin V–AbFluor™ 488/PI Apoptosis Detection Kit (Abbkine Scientific, Wuhan, China) following the manufacturer’s protocol. Stained cells were analyzed using an CytoFLEX flow cytometer (Beckman Coulter Life Sciences, Indianapolis, IN, USA), and apoptotic populations were quantified with FlowJo™ v10 (FlowJo LLC, Ashland, OR, USA).

### 2.4. Wound Healing and Transwell Invasion Assay

TE-1 and Eca-109 cells were plated in 6-well culture plates and cultured until approximately 80% confluence was reached. A sterile 1 mL pipette tip was then used to generate a linear scratch at the center of each well. After gentle washing with PBS to remove non-adherent cells, the remaining attached cells were cultured in medium containing 0.1% DMSO or apigenin (5, 10, or 20 μM) for 48 h. Cell migration into the wound area was photographed at 24 h using a microscope.

### 2.5. MicroRNA Transcriptome Profiling

Total RNA was mixed with 3′-ADT reagents, denatured at 70 °C for 2 min, and placed on ice. RNA ligation was performed by adding the manufacturer-provided ligation buffers, RNase inhibitor, ligases, and Ligation Enhancer Mix, followed by incubation at 37 °C for 1 h. After cooling on ice, 1 µL RT primer was added and briefly cycled for primer binding. For 5′-adapter ligations, the corresponding ligation buffer, RNase inhibitor, 5′-ADT, and RNA ligase were added and incubated again at 37 °C for 1 h. Reverse transcription was then carried out using RT Buffer Mix, RNase inhibitor, and reverse transcriptase according to standard conditions. The resulting cDNA was amplified using PCR Master Mix, a universal primer, and an index primer. Library size was checked on a 6% PAGE gel (Yamei Biotechnology Co., Ltd., Hangzhou, China), where successful libraries produced a clear ~140 bp band that was excised and purified. The library quality was confirmed by Bioanalyzer and qPCR, and sequencing was performed on the Illumina NovaSeq™ X Plus platform (SE50, ~10 M reads/sample). Processed reads were filtered, length-screened, and aligned to miRBase to identify known and novel miRNAs. Differential expression, target-gene prediction, and functional enrichment analyses were used to characterize miRNA profiles across groups. All miRNA sequencing was carried out by Hangzhou Astrocyte Technology Co., Ltd. (Hangzhou, China).

### 2.6. Bioinformatics and Target Gene Prediction

After quality filtering and length selection, clean miRNA-seq reads were aligned to the miRBase database to identify known miRNAs and predict novel candidates. Small RNAs were classified and annotated, and potential base-editing events were examined. Differential miRNA expression between the apigenin-treated and DMSO groups was then evaluated.

Differential miRNA expression was visualized using volcano plots generated in R. Read counts were normalized using log_2_ transformation, and miRNAs with a log_2_ fold-change > 1.0 and *p* < 0.05 were considered significantly altered.

To identify potential targets of the significantly dysregulated miRNAs, target prediction was conducted using miRanda (v3.3a) and RNAhybrid (v2.1.2). Each miRNA set was analyzed independently by both algorithms, and the intersecting genes were designated as the final predicted targets. When no overlap was observed, the union of predictions from the two tools was used instead.

Gene Ontology (GO) enrichment analysis was conducted to evaluate the biological processes, cellular components, and molecular functions associated with the predicted targets by using R package clusterProfiler v4.10.1. Enrichment significance was evaluated using FDR-corrected *p*-values, with FDR ≤ 0.05 as the threshold. KEGG pathway analysis was subsequently conducted to identify significantly enriched metabolic and signaling pathways by using, R package, clusterProfiler v4.10.1, also using FDR ≤ 0.05 to determine statistical significance.

### 2.7. RNA Isolation and Quantitative Real-Time PCR (qRT-PCR)

Following a 48 h exposure to 20 µM apigenin or 0.1% DMSO, TE-1 and Eca-109 cells were harvested for qRT-PCR analysis. Total RNA was isolated following the manufacturer’s protocol. Complementary DNA (cDNA) was then synthesized using the StarScript III All-in-One RT Mix with gDNA Remover (GenStar, A230-10, GenStar BioSolutions Co., Ltd., Beijing, China) for mRNA, and the StarScript III miRNA RT Kit with poly(A) tailing (GenStar, A238-02, GenStar BioSolutions Co., Ltd., Beijing, China) for miRNA. Expression levels of hsa-miRNAs and their corresponding target genes were measured using SYBR Green PCR Master Mix2×RealStar Fast SYBR qPCR Mix (GenStar A301-10, GenStar BioSolutions Co., Ltd., Beijing, China) on a Bio-Rad CFX Manager real-time PCR system. U6 was used as the internal control for miRNA detection, while GAPDH served as the reference gene for mRNA assays.

### 2.8. Statistical Analysis

The experiments were conducted in triplicate, and data are expressed as the mean ± standard deviation (SD). Statistical analyses were carried out using GraphPad Prism 8.0.1, applying one-way ANOVA or unpaired *t*-tests where appropriate. A *p*-value of less than 0.05 was considered indicative of statistical significance.

## 3. Results

### 3.1. Apigenin Suppresses the Proliferation of TE-1 and Eca-109 Cells

**Figure 1:**
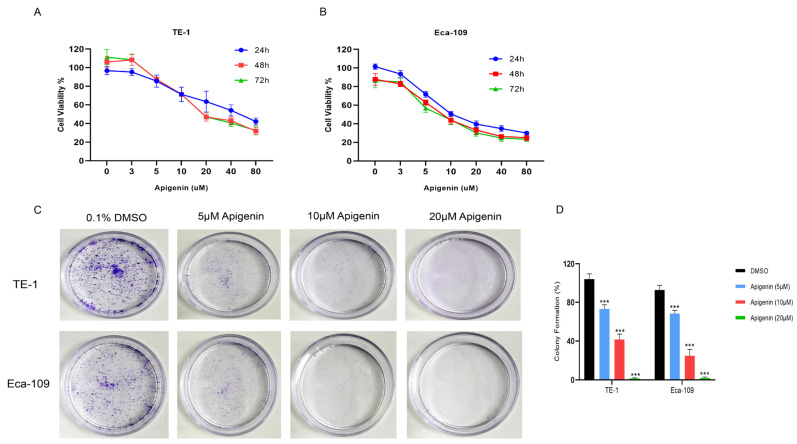
(**A**,**B**) Cell viability assay: TE-1 and Eca-109 cells were treated with increasing concentrations of apigenin (3, 5, 10, and 20 µM) for 72 h, and cell viability was measured using the CCK-8 assay. Apigenin significantly inhibited cell proliferation in a dose-dependent manner compared with the control group (0.1% DMSO). Data are presented as mean ± SD from three independent experiments. Statistical analysis was performed using two-way ANOVA followed by Tukey’s multiple-comparisons test in GraphPad Prism. *p* < 0.05 was considered statistically significant. (**C**) Colony formation assay: The ability of cells to form colonies after apigenin treatment was evaluated. Both the number and size of colonies were markedly reduced in apigenin-treated groups, indicating suppressed long-term proliferative capacity. (**D**) Results are shown as mean ± SD from three independent experiments. Two-way ANOVA was performed to analyze differences between groups, with Tukey’s post hoc test using GraphPad Prism. Statistical significance was defined as *p* < 0.05. *** *p* < 0.001 compared with the DMSO control group.

To evaluate the antiproliferative effects of apigenin on ESCC cell lines, i.e., TE-1 and Eca-109, they were treated with various concentrations of apigenin (3, 5, 10, 20, 40, and 80 µM) for 24, 48, and 72 h. Apigenin significantly inhibited cell proliferation in a dose-dependent manner (*p* < 0.05) (Figure 1A,B). The IC^50^ values of apigenin in TE-1 and Eca-109 cells were approximately 10 and 19, respectively. To evaluate the impact of apigenin on the clonogenic potential of TE-1 and Eca-109 cells, equal numbers of viable cells pretreated with either apigenin or DMSO were replated at low density. After 10 days of incubation, colonies were fixed, visualized, and quantified under a microscope. Apigenin markedly decreased both the number and size of colonies in a dose-dependent manner as compared with the control group (*p* < 0.01) (Figure 1C). These findings indicate that apigenin effectively suppresses the growth and clonogenic capacity of TE-1 and Eca-109 cells.

### 3.2. Apigenin Attenuated Wound Healing in TE-1 and Eca-109 Cells

**Figure 2:**
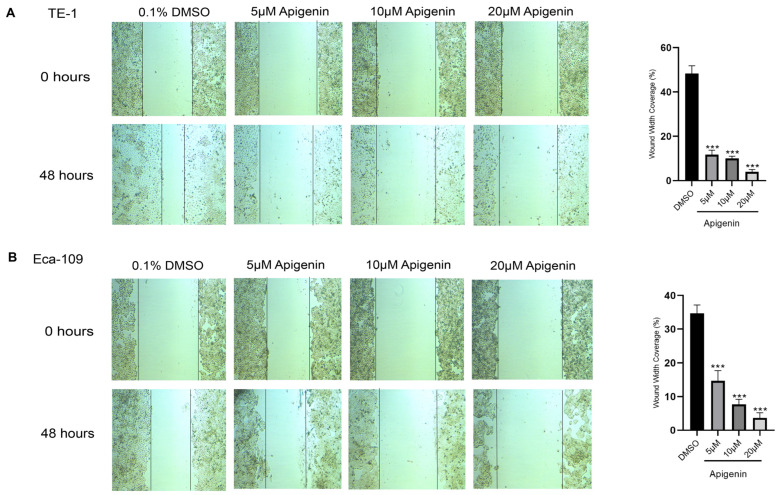
The migratory capacity of TE-1 (**A**) and Eca-109 (**B**) cells following apigenin treatment was assessed using a wound-healing assay. Cells were treated with apigenin (5, 10, and 20 µM) or vehicle control (0.1% DMSO), and wound closure was monitored at 0 and 48 h. Representative images are shown, and quantitative analysis of wound closure is presented as a percentage. Apigenin treatment significantly inhibited cell migration in a concentration-dependent manner. Data are expressed as mean ± SD from three independent experiments. Statistical analysis was performed using one-way ANOVA followed by Dunnett’s multiple-comparisons test in GraphPad Prism. *p* < 0.05 was considered statistically significant. *** *p* < 0.001 compared with the DMSO control group.

To further investigate the inhibitory effects of apigenin, we performed wound-healing assays to assess its impact on ESCC cell migration. Apigenin treatment noticeably slowed gap closure in TE-1 and Eca-109 cells at concentrations of 5, 10, and 20 µM (Figure 2A,B). The observed reduction in wound closure following apigenin treatment may be influenced by its anti-proliferative effects, indicating that the decrease likely reflects a combination of reduced cell proliferation and impaired migration rather than migration alone.

### 3.3. Apigenin Induces Apoptosis in ESCC Cells

**Figure 3:**
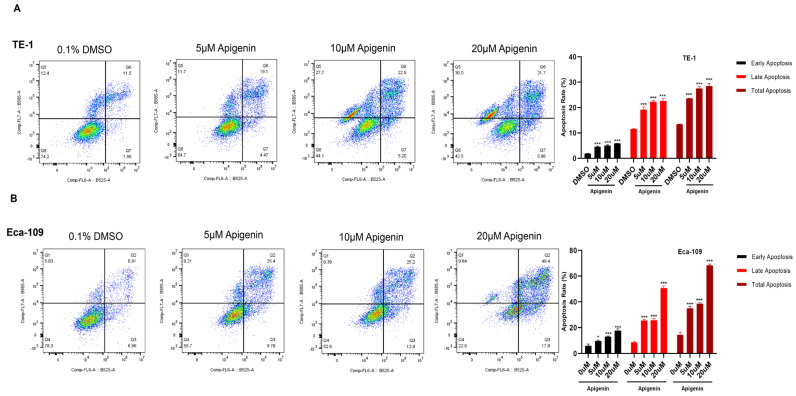
(**A**,**B**) Apigenin induces apoptosis in TE-1 and Eca-109 cells. Cells were treated with apigenin (5, 10, and 20 µM) for 48 h, and apoptosis was detected by Annexin V-AbFluor™ 488/PI Dual Staining Cell Apoptosis Detection Kit. Representative flow cytometry dot plots are shown, in which the color scale represents cell density (blue indicates low density and red indicates high density). Apigenin significantly increased apoptotic cell populations in both cell lines in a concentration-dependent manner. Quantitative form of data shown as early apoptosis, late apoptosis, and total apoptosis %. Results are presented as mean ± SD. Group differences were evaluated using two-way ANOVA with Tukey’s post hoc multiple-comparisons test performed in GraphPad Prism. Statistical significance was defined as *p* < 0.05. *p* < 0.05 was considered statistically significant. * *p* < 0.05, and *** *p* < 0.001 compared with the DMSO control group.

To determine whether apigenin influences apoptosis in ESCC cells, we employed the Annexin V–AbFluor™ 488/PI dual-staining apoptosis detection assay. Exposure to apigenin at concentrations of 5, 10, and 20 μM along with 0.1% DMSO control for 48 h, resulted in a marked increase in both early and late apoptotic populations in TE-1 and Eca-109 cells. Overall, our findings indicate that apigenin significantly enhances apoptosis in ESCC cells in a concentration-dependent manner (Figure 3A,B).

### 3.4. Annotation Total and Uniquely Mapped Reads from TE-1 and Eca-109 Cells

**Figure 4:**
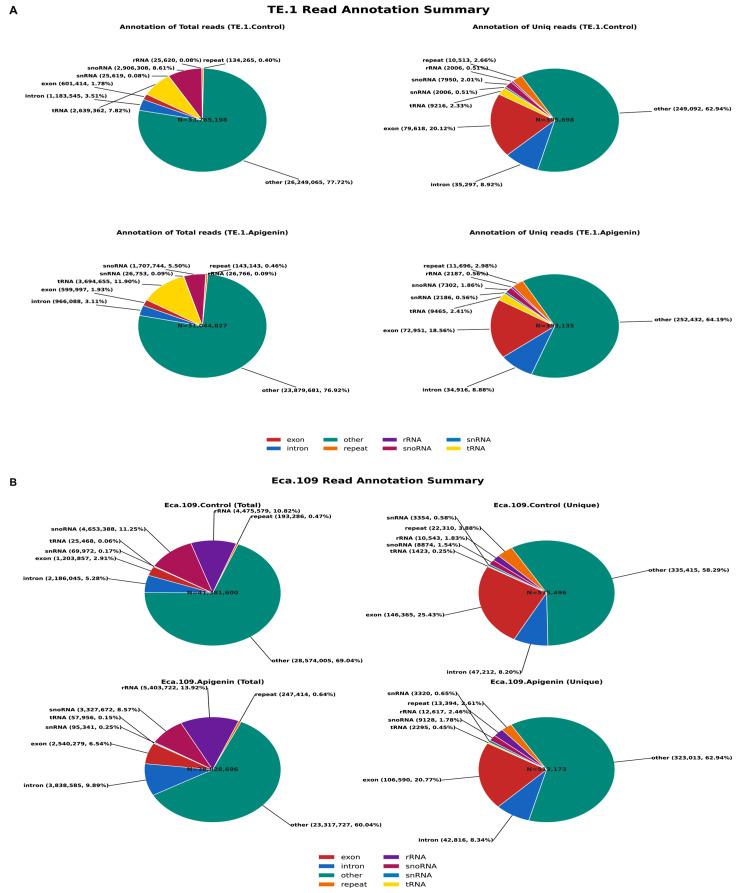
(**A**) Read annotation summary of TE.1 cells under control and Apigenin-treated conditions. (**A**) TE-1 cells. Pie charts show the distribution of total reads (**left**) and uniquely mapped reads (**right**) in control and apigenin-treated cells. Most total reads were classified as unannotated, with tRNA and snoRNA as the predominant annotated categories. Apigenin increased tRNA-derived reads and reduced snoRNA-associated reads, while uniquely mapped read distributions remained similar between groups, with enrichment in exonic and intronic regions. (**B**) Eca-109 cells. Pie charts illustrate the distribution of total reads (**left**) and uniquely mapped reads (**right**) in control and apigenin-treated cells. Apigenin reduced unannotated reads and increased rRNA-, exon-, and intron-derived reads in the total read pool, accompanied by decreased snoRNA levels. The uniquely mapped read profiles were largely unchanged, with exonic and intronic regions remaining dominant.

The annotation profiles of sequencing reads from TE-1 cell samples of DMSO and Apigenin are presented in Figure 4A. In both groups, most total mapped reads fell into the ‘other’ category, with tRNA- and snoRNA-derived reads comprising the major annotated classes. Apigenin treatment increased the proportion of tRNA-related reads while reducing snoRNA reads relative to DMSO. The uniquely mapped read distribution showed similar overall patterns across conditions, with consistent enrichment in exonic and intronic regions. However, shifts in tRNA, snoRNA, and repeat-derived categories suggest that apigenin alters the composition of total small RNA populations, while the genomic distribution of uniquely mapped reads remains largely stable.

Similarly, read annotation profiles of Eca-109 cells under DMSO and Apigenin-treated conditions are summarized in Figure 4B. In the total read population, the DMSO group showed a predominance of reads mapping to the ‘other’ category, with substantial contributions from rRNA and snoRNA. In contrast, the Apigenin-treated group showed a decreased proportion of ‘other’ reads, accompanied by increased rRNA-, exon-, and intron-derived reads, and a reduction in snoRNA abundance. Whereas, uniquely mapped reads displayed broadly similar distributions between groups, with exonic and intronic regions remaining the dominant categories. Although minor shifts were noted in tRNA, snoRNA, and repeat-derived reads, the overall profile of uniquely aligned reads was largely preserved. This indicates that apigenin alters the composition of total small RNA reads in Eca-109 cells while maintaining a stable distribution of uniquely mapped genomic features.

### 3.5. Apigenin Alters miRNA Expression Profiles in TE-1 and Eca-109 Cells

**Figure 5:**
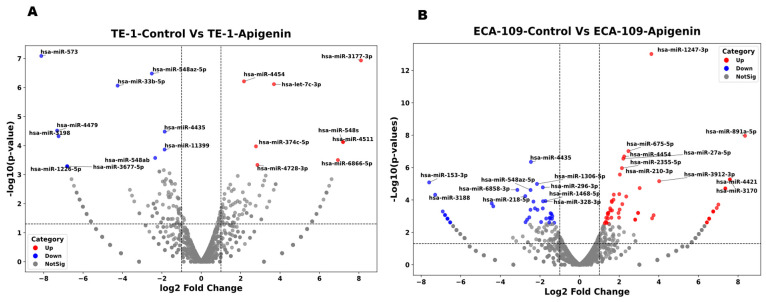
(**A**) Volcano map of the differential miRNA expression. TE-1 cells (TE-1 DMSO vs. TE-1 Apigenin). Each point represents an individual miRNA plotted by log_2_ fold change and −log_10_(*p*-value). Red and blue dots indicate significantly upregulated and downregulated miRNAs, respectively, while gray dots denote non-significant changes. Dashed lines indicate the thresholds of |log_2_ fold change| > 1 and *p* < 0.05. Labeled miRNAs indicate the most significantly dysregulated candidates. (**B**) Volcano plot of differentially expressed miRNAs between ECA-109 control and Apigenin-treated cells. Each point represents an individual miRNA plotted by log_2_ fold change versus −log_10_(*p*-value). Red and blue dots indicate significantly upregulated and downregulated miRNAs, respectively, while gray dots denote non-significant changes. Dashed lines indicate the thresholds of |log_2_ fold change| > 1 and *p* < 0.05. Labeled miRNAs represent the most significantly dysregulated transcripts following apigenin treatment, demonstrating substantial modulation of miRNA expression in Eca-109 cells.

To characterize miRNA expression, high-throughput RNA sequencing (RNA-seq) was employed in TE-1 cells, i.e., DMSO and Apigenin-treated groups. A total of 20 miRNAs were found to be differentially expressed, including 8 upregulated and 12 downregulated species Volcano plot clearly distinguished apigenin-treated cells from controls, and the top 20 differentially expressed miRNAs (Figure 5A). Several miRNAs exhibited significant dysregulation upon Apigenin exposure; however, hsa-miR-3177-3p, hsa-miR-4454, hsa-let-7c-3p, and hsa-miR-4511 were markedly upregulated, while hsa-miR-573, hsa-miR-33b-5p, hsa-miR-548az-5p, and hsa-miR-4479 were significantly downregulated.

On the other hand, the miRNA expression profiles of Eca-109 cells exhibited a comprehensive transcriptomic data following apigenin treatment. A total of 92 miRNAs were found to be differentially expressed, including 54 upregulated and 38 downregulated species. Volcano plot clearly distinguished apigenin-treated cells from controls, and the top 92 differentially expressed miRNAs (Figure 5B). Apigenin treatment markedly upregulated, including hsa-miR-1247-3p, hsa-miR-891a-5p, hsa-miR-210-3p, hsa-miR-675-5p, and hsa-miR-27a-5p, while others such as hsa-miR-153-3p, hsa-miR-548az-5p, hsa-miR-218-5p, and hsa-miR-4435, were notably downregulated.

### 3.6. Confirmation of Differential miRNA and Target Gene Expression via qRT-PCR

**Figure 6:**
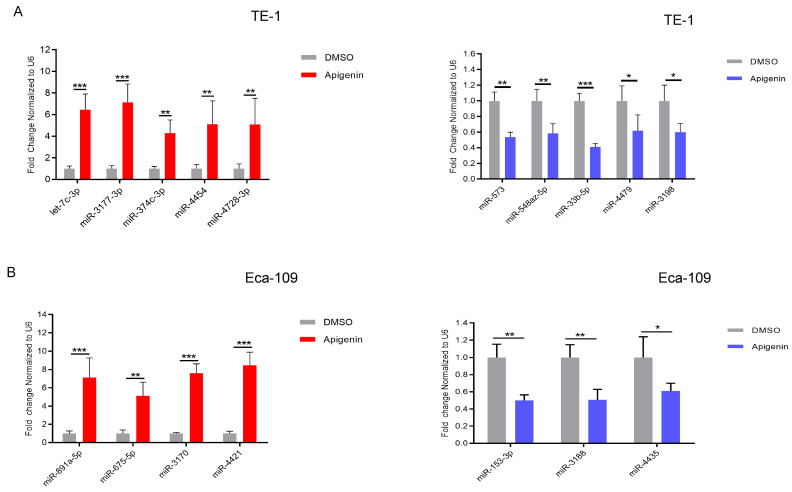

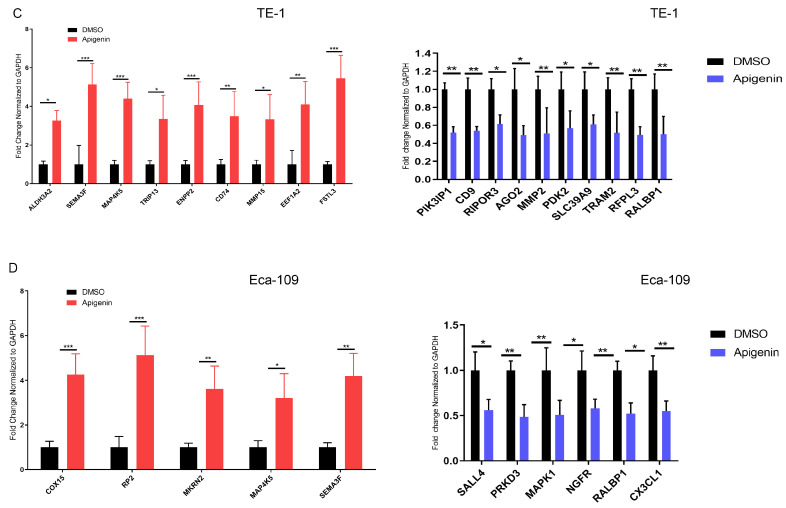
Confirmation of Altered miRNAs and Their Target Genes in apigenin-treated ESCC cells. The miRNAs exhibiting altered expression and their corresponding target genes identified by RNA sequencing were validated by RT–qPCR in apigenin-treated and control (0.1% DMSO) TE-1 and Eca-109 cells. (**A**) RT–qPCR validation of selected miRNAs in TE-1 cells. (**B**) RT–qPCR validation of 7 selected miRNAs in Eca-109 cells. (**C**,**D**) RT–qPCR analysis confirming the differential expression of representative target genes in TE-1 and Eca-109 cells, respectively. Data represent the mean ± SD of three independent experiments. Statistical evaluation was conducted by two-way ANOVA followed by Šídák’s multiple-comparisons test, with significance defined as * *p* < 0.05, ** *p* < 0.01, and *** *p* < 0.001 compared with the control group.

To verify the RNA sequencing data, selected miRNAs in TE-1 cells were further examined by quantitative real-time PCR (qRT-PCR) after Apigenin treatment, along with a DMSO control. The qRT-PCR results largely corroborated the RNA-seq data. Specifically, hsa-let-7c-3p, hsa-miR-3177-3p, hsa-miR-374c-3p, hsa-miR-4454, and hsa-miR-4728-3p were significantly upregulated following apigenin treatment, whereas hsa-miR-573, hsa-miR-548az-5p, hsa-miR-33b-5p, hsa-miR-4479, and hsa-miR-3198 were markedly downregulated. (Figure 6A). In TE-1 cells, the qPCR mRNA expression analysis revealed that Aldehyde dehydrogenase 3 family member A2 (*ALDH3A2*), Semaphorin 3F (*SEMA3F*), Mitogen-activated protein kinase 5 (*MAP4K5*), Thyroid hormone receptor interactor 13 (*TRIP13*), Ectonucleotide pyrophosphatase/phosphodiesterase 2 (*ENPP2*), CD74 molecule, major histocompatibility complex class II invariant chain (*CD74*), Matrix metallopeptidase 15 (MMP15), Eukaryotic translation elongation factor 1 alpha 2 (*EEF1A2*), and Follistatin-like 3 (*FSTL3*)were markedly upregulated following apigenin treatment compared with DMSO-treated controls. In contrast, the expression level of phosphoinositide-3-kinase interacting protein 1 (*PIK3IP1*), CD9 molecule (*CD9*), Rho family–interacting cell polarization regulator 3 (*RIPOR3*), Argonaute RISC catalytic component 2 (*AGO2*), matrix metallopeptidase 2 (*MMP2*), pyruvate dehydrogenase kinase 2 (*PDK2*), solute carrier family 39 member 9 (*SLC39A9*), translocation associated membrane protein 2 (*TRAM2*), ret finger protein-like 3 (*RFPL3*), and RalA binding protein 1 (RALBP1)were significantly downregulated in apigenin-treated TE-1 cells (Figure 6C).

In Eca-109 cells, apigenin treatment resulted in significant upregulation of hsa-miR-891-5p, hsa-miR-675-5p, hsa-miR-3170, hsa-miR-4421, and hsa-miR-3912. In contrast, hsa-miR-153, hsa-miR-3188, hsa-miR-548az-5p, and hsa-miR-4435 were among the most significantly downregulated miRNAs following apigenin exposure. (Figure 6B). Along with these miRNAs, in Eca-109 cells, apigenin treatment resulted in increased expression of certain mRNAs such as Cytochrome c Oxidase Assembly Factor 15 (*COX15*), Retinitis Pigmentosa 2 (*RP2*), Makorin Ring Finger Protein 2 (*MKRN2*), and Semaphorin 3F (*SEMA3F*), whereas Sal-like Protein 4 (*SALL4*), Protein Kinase D3 (*PRKD3*), Mitogen-Activated Protein Kinase 1 (*MAPK1*), Nerve Growth Factor Receptor (*NGFR*), RalA Binding Protein 1 (*RALBP1*), and C-X3-C Motif Chemokine Ligand 1 (*CX3CL1*) were notably downregulated compared with the control group (Figure 6D). These could be the potential targets of the miRNAs.

The mRNAs validated by bioinformatic analysis and RT–qPCR were visualized in miRNA–mRNA interaction network plots, which are provided in the Supplementary Data Figures S1 and S2.

### 3.7. GO Functional Enrichment Analysis of miRNA-Associated Target Genes

**Figure 7:**
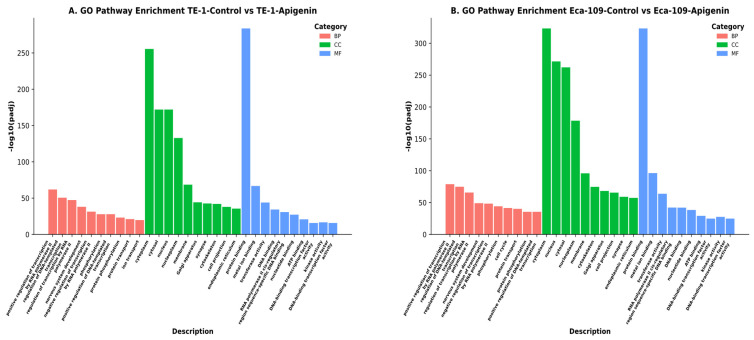
GO enrichment analysis was conducted for genes with altered expression in ESCC cells following apigenin treatment. GO enrichment analysis was conducted to identify biological processes (BP), cellular components (CC), and molecular functions (MF) that were significantly enriched between apigenin-treated and DMSO-treated control cells in TE-1 (**A**) and Eca-109 (**B**) cell lines. The bar plots display the top enriched GO terms, with the x-axis indicating GO term descriptions and the y-axis representing enrichment significance. Enrichment significance was calculated using a hypergeometric test and adjusted for multiple comparisons using the Benjamini–Hochberg false discovery rate (−log_10_ adjusted *p*-value). In both cell lines, enriched BP terms were mainly related to transcriptional regulation and phosphorylation, CC terms were predominantly associated with the cytoplasm, nucleus, and membrane-related compartments, and MF terms were largely enriched in protein binding, metal ion binding, and transcription factor-associated activities.

Gene Ontology (GO) analysis of genes differentially expressed between apigenin-treated and DMSO-treated TE-1 cells demonstrated significant enrichment in Biological Process (BP), Cellular Component (CC), and Molecular Function (MF) categories. Enriched BP terms were mainly associated with transcriptional regulation, signal transduction, and protein phosphorylation. CC analysis indicated predominant localization of affected genes to the cytoplasm, nucleus, membrane, endoplasmic reticulum, Golgi apparatus, and cytoskeleton. MF terms were primarily related to protein and DNA binding, kinase and transferase activities, ATP binding, and transcription factor activity. Overall, these results suggest that apigenin induces broad functional alterations in gene regulation, signaling, and subcellular organization in TE-1 esophageal squamous cell carcinoma cells (Figure 7A).

Gene Ontology (GO) enrichment analysis of genes showing differential expression between apigenin-treated and DMSO-treated Eca-109 cells revealed significant enrichment in BP, CC, and MF categories. BP terms were mainly associated with transcriptional regulation, RNA polymerase II-dependent transcription, protein phosphorylation, and signal transduction. CC analysis showed predominant localization to the cytoplasm, nucleus, membrane, Golgi apparatus, endoplasmic reticulum, and cytoskeleton. MF terms were primarily related to protein and DNA binding, transferase and kinase activities, ATP binding, and transcription factor activity, indicating that apigenin broadly affects gene regulation and intracellular signaling in Eca-109 cells (Figure 7B).

Overall, the GO enrichment patterns were largely consistent between the two cell lines, supporting the notion that apigenin induces comparable functional reprogramming at the molecular and cellular levels in ESCC cells.

### 3.8. KEGG Pathway Analysis of Altered miRNAs and Their Predicted Target Genes

To determine the biological significance of the miRNA–gene interactions, Kyoto Encyclopedia of Genes and Genomes (KEGG) was utilized for pathway enrichment analysis.

**Figure 8:**
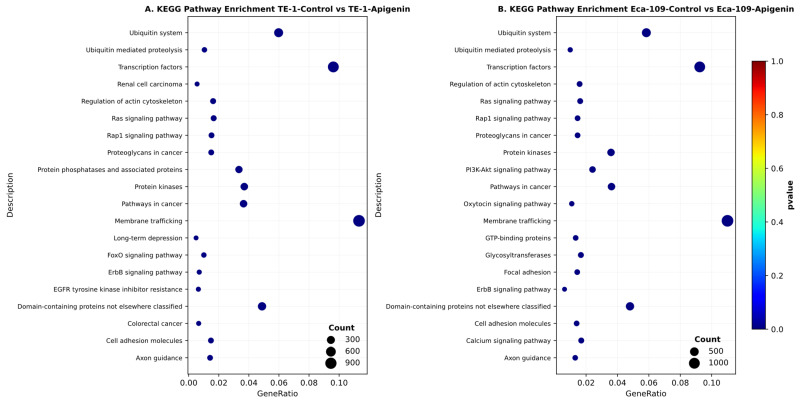
Analysis of KEGG pathway enrichment for genes with altered expression in ESCC cells treated with apigenin. Pathway enrichment analysis using KEGG was performed to compare apigenin-treated and control (DMSO-treated) (**A**) TE-1 cells (**B**) Eca-109 cells. The bubble plots display the top enriched KEGG pathways, with the x-axis representing the GeneRatio and the y-axis indicating pathway names. Bubble size corresponds to the number of genes enriched in each pathway, while color reflects the enrichment significance. Enrichment significance was calculated using a hypergeometric test and adjusted for multiple comparisons using the Benjamini–Hochberg false discovery rate (−log_10_ adjusted *p*-value). In both cell lines, apigenin treatment was mainly associated with pathways related to cancer signaling, protein kinases, ubiquitin-mediated processes, membrane trafficking, and cell adhesion signaling.

KEGG analysis of genes with altered expression between apigenin-treated and DMSO-treated TE-1 cells revealed significant enrichment of cancer- and signaling-related pathways. Notably enriched pathways included Pathways in cancer, Rat sarcoma (Ras), Ras-proximate-1 (Rap1), Erythroblastic leukemia viral oncogene homolog (ErbB), and Forkhead box O (FoxO) signaling, as well as pathways related to protein kinases, protein phosphatases, ubiquitin-mediated proteolysis, membrane trafficking, regulation of the actin cytoskeleton, and cell adhesion. These findings indicate that apigenin induces broad transcriptional alterations in TE-1 cells, particularly impacting oncogenic signaling, protein turnover, and cytoskeletal organization, which may underlie its anti-tumor effects in esophageal squamous cell carcinoma. These results suggest that apigenin treatment induces broad transcriptional changes in TE-1 cells, particularly affecting oncogenic signaling, protein turnover, and cellular structural dynamics, which may collectively contribute to its anti-tumor effects in esophageal squamous cell carcinoma. (Figure 8A).

KEGG-based enrichment analysis of differentially expressed genes in Eca-109 cells following apigenin treatment compared to DMSO controls indicated significant enrichment of cancer-related and signaling pathways, including the Pathways in Cancer, phosphoinositide 3-kinase–protein kinase B (PI3K–Akt), Ras, Rap1, and ErbB signaling pathways. Pathways involved in protein regulation and cellular dynamics, such as protein kinases, the ubiquitin system, ubiquitin-mediated proteolysis, and membrane trafficking, were also prominently enriched. In addition, pathways related to cell adhesion and cytoskeletal organization, including focal adhesion, regulation of the actin cytoskeleton, and axon guidance, were significantly represented. These findings indicate that apigenin induces broad transcriptional changes in Eca-109 cells, affecting oncogenic signaling, protein turnover, membrane transport, and cell–cell interactions, which may contribute to its anti-tumor effects in esophageal squamous cell carcinoma (Figure 8B).

Collectively, these results suggest that apigenin treatment induces extensive transcriptional reprogramming in both TE-1 and Eca-109 cells, particularly affecting oncogenic signaling cascades, protein turnover, membrane transport, and adhesion-related processes. These pathway alterations are consistent with the anti-proliferative and anti-tumor effects of apigenin in esophageal squamous cell carcinoma cells.

## 4. Discussion

Apigenin, a naturally occurring flavonoid found in various fruits and vegetables, has been shown to possess broad-spectrum anticancer properties in multiple malignancies by inhibiting cell proliferation, suppressing migration and invasion, and promoting apoptosis with relatively low toxicity in preclinical studies [18]. In this study, we demonstrated that apigenin markedly suppressed proliferation, colony formation, and wound healing capacity in human esophageal squamous cell carcinoma (ESCC) cell lines TE-1 and Eca-109. Consistent with previous reports in ESCC and other cancer types, apigenin significantly promoted apoptotic cell death, suggesting activation of intrinsic and/or extrinsic apoptosis pathways, as evidenced by increased apoptosis markers such as cleaved caspase-8 and cleaved PARP in treated cancer cells [7]. Moreover, apigenin treatment was accompanied by widespread alterations in miRNA expression and modulation of downstream target genes that are implicated in tumor progression and survival. These findings provide deeper mechanistic insight into the antitumor activity of apigenin in ESCC. Beyond ESCC, apigenin has been shown to regulate cancer-related miRNA networks and their target genes in hepatocellular carcinoma and colorectal cancer, linking miRNA modulation to its antitumor activity [9]. However, the data provided in this study were limited; therefore, our work extends these findings by implicating miRNA alterations as a mechanistic axis for apigenin-mediated suppression of ESCC progression, thereby strengthening the rationale for further exploration of apigenin as a complementary anticancer agent.

Apigenin is a flavonoid widely distributed in medicinal and dietary plants, including Matricaria chamomilla (chamomile), Petroselinum crispum (parsley), celery, onions, and citrus fruits, many of which have longstanding use in traditional medicine systems for their anti-inflammatory and health-promoting properties [19].Traditional remedies often demonstrate multi-target biological effects that cannot be fully explained by single protein targets, and miRNA-mediated regulation offers a molecular framework for these complex activities [20]. Natural compounds, including flavonoids, have been shown to modulate miRNA expression and influence gene networks involved in proliferation, apoptosis, and metastasis, which is increasingly recognized as a mechanism by which bioactive phytochemicals exert antitumor effects [21]. By integrating miRNA profiling with pathway analysis, studies of traditional medicine-derived agents like apigenin help bridge ethnopharmacological knowledge with modern molecular biology and may guide the development of multi-target therapeutic strategies based on miRNA regulatory networks.

The observed dose-dependent inhibition of cell viability and clonogenic growth is consistent with previous studies reporting that flavonoids suppress esophageal cancer progression by interfering with cell-cycle regulation and survival signaling pathways [22,23]. Moreover, apigenin significantly reduced wound healing in both ESCC cell lines, indicating impaired migratory capacity and anti-proliferative effects. This observation aligns with earlier reports demonstrating that apigenin inhibits migration and invasion in various cancer models by targeting cytoskeletal remodeling and adhesion-related pathways [24,25]. However, the wound-healing assay may be influenced by the anti-proliferative effects of apigenin, and therefore, reduced wound closure cannot be interpreted solely as a reflection of impaired migration. Notably, the marked increase in apoptotic cell populations, as confirmed by Annexin V/PI staining, indicates that apigenin not only halts proliferation but also actively triggers programmed cell death. Similar pro-apoptotic effects of apigenin have been reported in breast, colorectal, and hepatocellular carcinoma cells, supporting its broad anticancer potential [8,26].

The miRNAs are critical post-transcriptional regulators in ESCC, governing tumor initiation, progression, metastasis, and therapeutic response. In TE-1 cells, apigenin treatment resulted in significant differential expression of multiple miRNAs, including both upregulated and downregulated species. The miRNAs and target genes selected for qRT-PCR validation in this study were chosen based on a combination of statistical and biological considerations. We prioritized candidates showing robust differential expression with strong statistical support in our sequencing data and those implicated in cancer-related pathways from enrichment analyses, particularly pathways linked to cell proliferation, apoptosis, and metastasis. This approach aligns with other recent ESCC miRNA studies in which candidate miRNAs with consistent differential expression patterns and biological relevance were validated by qRT-PCR to support broader functional interpretation in clinical contexts. Where possible, priority was also given to miRNAs and target genes with previously reported roles in ESCC or related cancers, strengthening the biological relevance of our validation results.

In TE-1 cells, apigenin treatment resulted in the upregulation of multiple miRNAs that were incorporated into miRNA–mRNA regulatory networks. let-7c-3p was associated with the downregulation of its predicted targets *PIK3IP1*, *CD9*, and *RIPOR3*, as supported by target prediction analyses and qPCR validation (Supplementary Figure S1A. Similarly, miR-3177-3p was linked to *AGO2*, *MMP2*, and *PDK2*, as illustrated in Supplementary Figure S1B. In addition, miR-374c-3p targeted *SLC39A9*, while miR-4454 was associated with *TRAM2* and *RFPL3*, with all interactions validated by sequencing data and qPCR (Supplementary Figure S1A). Furthermore, miR-4728 was predicted to regulate *RALBP1*, as shown in Supplementary Figure S1A. Collectively, these data indicate that apigenin induces coordinated miRNA upregulation, contributing to the suppression of oncogenic gene expression in ESCC cells.

Our findings underscore the tumor-suppressive roles of specific miRNAs in cancer. Notably, miR-let-7c-5p has been reported to suppress invasion and migration in bladder cancer by targeting High mobility group AT-hook 2 (*HMGA2*), further supporting a broad tumor-suppressive role for let-7c family members [27,28]. In mucosal melanoma, reduced let-7c correlates with aggressive phenotypes, and restoration of let-7c inhibits tumor growth, invasion, and enhances chemotherapy sensitivity [29]. In hepatocellular carcinoma (HCC), epigenetic silencing of the let-7c cluster contributes to progression and metastasis, and let-7c-5p inhibits HCC cell proliferation and invasion by targeting Enhancer of zeste homolog 2 (*EZH2*), linking let-7c loss to malignant behaviors and poor prognosis [30,31]. The miR-3177-3p and miR-374c-3p remain largely uncharacterized in cancer, with minimal functional evidence published to date, indicating a gap in the current miRNA oncology literature.

The miR-4454 has context-dependent roles in cancer, such as in ovarian cancer models, miR-4454 is downregulated during metastatic progression, and its overexpression suppresses metastatic colonization in xenografts, suggesting tumor-suppressive activity within that context [32]. The miR-4728-3p has been implicated as a tumor suppressor in colorectal neoplasia associated with ulcerative colitis, where its downregulation correlates with neoplastic progression, and exogenous miR-4728-3p reduces focal adhesion signaling and invasive behavior in vitro [33]. This supports a potential suppressive role in specific cancer contexts, although additional studies are needed to clarify its functions across tumor types.

Conversely, several miRNAs were downregulated following apigenin treatment and were associated with increased expression of their predicted target genes. Notably, miR-573 was reduced alongside upregulation of *ALDH3A2* and *SEMA3F* (Supplementary Figure S2A). Similarly, miR-548az-5p and miR-33b-5p were downregulated and linked to *MAP4K5/TRIP13* and *ENPP2/CD74*, respectively. In addition, miR-4479 was associated with MMP15 and EEF1A2, while miR-3198 was predicted to target *FSTL3*.

Among the miRNAs analyzed, miR-573 shows the most consistent evidence of tumor-suppressive activity. It is frequently downregulated in pancreatic cancer, and its restoration inhibits cell proliferation, migration, invasion, and tumor growth through targeting oncogenic factors such as E2F transcription factor 3 (*E2F3*) and Tetraspanin 1 (*TSPAN1*) [34,35]. The miR-548az-5p has not yet been directly linked to tumor suppression in cancer models. However, it has been shown to negatively regulate cell proliferation and induce cellular senescence in non-cancer epithelial cells via targeting Katanin catalytic subunit A1-like 1 (*KATNAL1*), suggesting a potential growth-regulatory function that may be relevant in cancer contexts, pending further validation [36]. There is clear evidence that miR-33b-5p functions as a tumor suppressor in several malignancies. In renal cell carcinoma, miR-33b-5p is downregulated, and its re-expression suppresses proliferation and invasion and is associated with favorable clinical outcomes [37]. Similar tumor-inhibitory roles have been reported in gastric and colorectal cancers, indicating a broader anti-tumor function across cancer types [38,39].

For miR-4479 and miR-3198, the current literature provides limited functional evidence for direct roles in tumor suppression. miR-4479 has been detected in circulating exosomes from cancer patients and proposed as a potential biomarker, but mechanistic studies supporting a functional tumor-suppressive role are lacking [40]. Likewise, miR-3198 remains largely uncharacterized in the context of cancer biology.

Consistently, miRNA–mRNA network analysis identified *ALDH3A2*, *SEMA3F*, *MAP4K5*, and *TRIP13*, as well as *PIK3IP1*, *CD9*, *RIPOR3*, and *AGO2*, as key regulatory nodes in TE-1 cells, suggesting that apigenin reshapes the ESCC transcriptome through miRNA-directed regulation of oncogenic signaling. KEGG pathway enrichment analysis further revealed significant involvement of PI3K–Akt signaling, *MAPK* signaling, and cell adhesion-associated pathways, all of which play central roles in ESCC growth, survival, and metastasis and are tightly regulated by miRNA–mRNA interactions.

In Eca-109 cells, apigenin treatment led to the upregulation of several miRNAs accompanied by reduced expression of their predicted target genes. Specifically, miR-891 was increased and associated with downregulation of *SALL4* and *PRKD3*, while miR-675-5p upregulation correlated with decreased *MAPK1* and Nerve growth factor receptor (*NGFR*) expression. In addition, elevated miR-3170 and miR-4421 levels were linked to suppression of *RALBP1* and *CX3CL1*, respectively. Together, these results indicate that apigenin modulates miRNA-mediated regulatory networks in ESCC cells, contributing to the inhibition of oncogenic signaling pathways.

Among the miRNAs examined, miR-675-5p exhibits context-dependent roles in cancer rather than a uniform tumor-suppressive function. In lung cancer, decreased miR-675-5p expression has been associated with enhanced tumor progression and metastasis, suggesting a tumor-suppressive role in this context [41]. In contrast, studies in colorectal cancer report lower miR-675-5p levels in tumor tissues, while paradoxically associating higher expression with poorer clinical outcomes, underscoring its complex and cancer-type-specific functions [42]. For miR-891a-5p, emerging evidence indicates that miR-891 family members play significant roles in cancer progression. For instance, miR-891a-5p is upregulated in non-small cell lung cancer and promotes proliferation and metastasis by targeting Homeobox A5 (*HOXA5*), while in hormone receptor-positive breast cancer, low miR-891a-5p expression correlates with poorer prognosis, and its overexpression suppresses cell proliferation and migration [43,44]. Previous research has implicated miR-3170 in cancer biology, where its expression contributed to a prognostic miRNA signature in sarcoma patients, indicating its potential relevance in tumor progression and clinical outcomes [45]. Although functional studies directly characterizing miR-4421 in cancer are limited, genetic variation affecting the miR-4421 binding site in the Endoplasmic reticulum protein 29 (*ERP29*) 3′-UTR has been linked to cancer risk and prognosis in oropharyngeal squamous cell carcinoma, suggesting that miR-4421 may influence tumor biology via modulation of target gene expression [46].

In Eca-109 cells, apigenin treatment resulted in the downregulation of several miRNAs with concomitant upregulation of their predicted target genes. Specifically, reduced miR-153-5p expression was associated with increased *COX15* and *RP2* levels, while downregulation of miR-3198 and miR-4435 correlated with elevated expression of *MKRN2* and *SEMA3F*, respectively. These data indicate that apigenin promotes selective miRNA downregulation, leading to derepression of downstream targets.

Among the miRNAs analyzed, miR-153-5p displays a context-dependent role in cancer, such as a study that miR-153-5p has been reported to function as a tumor-suppressive miRNA in cancer, where its overexpression inhibits cell proliferation, migration, and Epithelial–Mesenchymal Transition (EMT)-related phenotypes by targeting oncogenic factors such as Wilms’ tumor 1 (*WT1*) in esophageal squamous cell carcinoma [47]. Most studies support a tumor-suppressive function, as miR-153-5p inhibits proliferation, invasion, epithelial–mesenchymal transition, and stemness across multiple cancer types [48]. In triple-negative breast cancer, miR-153-5p enhances paclitaxel sensitivity and promotes apoptosis, further supporting its suppressive role [49]. However, in clear-cell renal cell carcinoma, miR-153-5p has been reported to promote tumor growth and metastasis, underscoring its cancer-type-specific behavior [50].

Although functional studies directly assessing the tumor-suppressive effects of miR-3198 are limited, clinical profiling has shown that miR-3198 expression is significantly dysregulated in several cancers, including hepatocellular carcinoma and nasopharyngeal carcinoma, suggesting its involvement in cancer progression and potential regulatory roles [51].

The miR-4435 is downregulated in malignant breast tumors compared with normal tissue, suggesting a potential tumor-suppressive association, although functional validation is lacking [52]. Although direct evidence for a tumor-suppressive role of miR-4435 is limited, miR-4435 has been reported to be upregulated in colorectal cancer, where it negatively regulates the tumor suppressor gene Tissue inhibitor of metalloproteinases 3 (*TIMP3*), and inhibition of miR-4435 increases *TIMP3* expression and decreases proliferation and migration of cancer cells [53].

Correspondingly, miRNA–mRNA network analysis in Eca-109 cells identified *COX15*, *RP2*, *MKRN2*, and *SEMA3F*, as well as *SALL4*, *PRKD3*, *MAPK1*, *NGFR*, *RALBP1*, and *CX3CL1*, as key regulatory nodes. GO and KEGG analyses revealed enrichment of transcriptional regulation, phosphorylation-related processes, and oncogenic signaling pathways, including PI3K–Akt, *MAPK*, and cell adhesion signaling. These results suggest that apigenin suppresses ESCC progression by reprogramming miRNA-driven regulatory networks and attenuating key oncogenic pathways.

A notable observation in our study is that the miRNA expression profiles altered by apigenin differ substantially between TE-1 and Eca-109 cells. Esophageal squamous cell carcinoma (ESCC) is characterized by considerable molecular heterogeneity, encompassing differences in differentiation status, genetic background, chromatin accessibility, and baseline transcriptomic landscapes across both tumors and cell line models, which has been shown to affect pathway activity and cellular phenotypes in ESCC contexts. Such intrinsic variation likely contributes to the divergent miRNA responses observed here, as baseline miRNA expression and downstream regulatory networks can vary markedly even among commonly used ESCC lines. This heterogeneity is consistent with evidence from multi-omics and single-cell analyses demonstrating distinct molecular subtypes and regulatory states within ESCC that influence gene expression programs [54,55]. Therefore, while the canonical inverse relationships we focus on are reproducible across both cell lines, the magnitude and specific miRNA changes must be interpreted within the context of underlying cellular differences. Therefore, these cell line–specific responses illustrate broader tumor heterogeneity and may affect the generalizability of mechanistic insights to other ESCC models or clinical samples.

Furthermore, the selected miRNAs consistently followed the canonical miRNA–mRNA regulatory pattern in both ESCC cell lines. Specifically, changes in miRNA expression were inversely correlated with the expression of their corresponding target mRNAs, supporting the classical model of miRNA-mediated post-transcriptional regulation. The consistency of this canonical relationship across both TE-1 and Eca-109 cells strengthens the reliability of the selected miRNA–mRNA interactions and underscores their potential biological relevance in the context of apigenin treatment. In the canonical miRNA regulatory pathway, mature miRNAs loaded into Argonaute complexes bind complementary sites in the 3′ untranslated region of target mRNAs, resulting in translational repression and/or mRNA destabilization, so that upregulation of a canonical miRNA is typically associated with downregulation of its target mRNA [56,57]. By contrast, non-canonical miRNA pathways encompass alternative processing routes and target interactions outside classical seed-matched 3′ UTR sites, and can produce regulatory outcomes that do not follow a simple inverse expression pattern or may even involve stabilization or activation of transcripts [58]. Thus, an inverse correlation between miRNA and mRNA levels is characteristic of canonical miRNA function, whereas deviations from this pattern can reflect non-canonical mechanisms. We also observed a subset of miRNAs that showed non-canonical expression patterns relative to their predicted target genes following apigenin treatment. These miRNAs were not included in the final analysis to maintain consistency and to focus on regulatory relationships that follow the classical inverse expression pattern typical of canonical miRNA-mediated regulation. By concentrating on these well-established interactions, we aimed to minimize interpretational complexity and present clearer regulatory networks. We nevertheless recognize that non-canonical miRNA functions may also play a role in the cellular response to apigenin and merit further investigation in future studies.

Despite the comprehensive bioinformatic analyses and qPCR validation performed in this study, we acknowledge that our findings mainly describe associations between apigenin treatment, altered miRNA expression, and downstream target genes, rather than direct mechanistic causality. While the integration of differential expression analysis, pathway enrichment, and experimental validation supports the biological relevance of our results, the specific contribution of individual miRNAs to the observed effects on proliferation, apoptosis, and migration has not been directly demonstrated. In future studies, we plan to focus on a limited number of the most significantly dysregulated miRNAs together with their validated target mRNAs for functional analysis. To address these limitations, future research should focus on in vivo validation using animal models or patient-derived tissues by using miRNA mimics and inhibitors in combination with Apigenin to confirm the observed effects under physiological conditions. These Clinical correlation studies will allow us to more precisely define the miRNA-mediated signaling pathways involved in the anti-metastatic activity of apigenin in ESCC, thereby strengthening causal interpretation and mechanistic insight.

Collectively, our findings support a mechanistic model in which apigenin alters miRNA expression to regulate downstream targets that converge on critical signaling pathways, including PI3K–Akt, MAPK, and membrane-associated ubiquitin systems, thereby shifting the cellular balance from proliferation toward apoptosis and inhibiting ESCC cell invasion. Future studies should validate direct miRNA–mRNA interactions using luciferase reporter assays and gain- or loss-of-function approaches. Furthermore, investigating the clinical relevance of the identified miRNAs and exploring combinatorial strategies involving apigenin and conventional chemotherapeutics may enhance therapeutic efficacy.

In summary, apigenin exerts potent antitumor effects in ESCC by modulating miRNA expression and downstream oncogenic signaling networks that govern proliferation, apoptosis, and invasion. These findings provide new mechanistic insight into the anticancer activity of apigenin and support its potential as a therapeutic or adjuvant agent for ESCC treatment.

## 5. Conclusions

This study demonstrates that apigenin effectively suppresses malignant phenotypes in human esophageal squamous cell carcinoma (ESCC) cells, including TE-1 and Eca-109, by inhibiting proliferation, colony formation, migration, and invasion, while inducing apoptosis in a dose-dependent manner. Integrated miRNA transcriptome and bioinformatic analyses further revealed that apigenin remodels miRNA–mRNA regulatory networks involved in Protein kinases, apoptosis, and metastasis-associated signaling, identifying several key genes as potential molecular targets. Collectively, these findings provide mechanistic insight into the anticancer activity of apigenin and support its potential as a therapeutic or adjuvant agent for ESCC treatment.

## Data Availability

The original sequencing data can be found under the Read Archive (SRA) submission: SUB15908085. Further inquiries can be directed to the corresponding author or first author.

## Supplementary Materials

The following supporting information can be downloaded at: https://www.mdpi.com/article/10.3390/biom16030366/s1, Figure S1A: Upregulated miRNAs following apigenin treatment in TE-1 and their potential validated target genes; Figure S1B: Downregulated miRNAs following apigenin treatment in TE-1 cells and their validated target genes; Figure S2A: Upregulated miRNAs following apigenin treatment in Eca-109 cells and their validated target genes; Figure S2B: Downregulated miRNAs following apigenin treatment in Eca-109 cells and their validated target genes; Table S1: qRT-PCR primer sequences used to test miRNA expression; Table S2: Primer sequences used for qRT-PCR of mRNA/gene.
