# Supplementary material for: Apigenin Inhibits the Growth of Esophageal Squamous Cell Carcinoma (ESCC) Cells by Harnessing the Expression of MicroRNAs
Source: Biomolecules. 2026 Feb 28;16(3):366. doi: 10.3390/biom16030366 (PMC13023640; doi:10.3390/biom16030366)
Supplement: Supplementary file 1 [file biomolecules-16-00366-s001.zip › biomolecules-4104293-supplementary.pdf]

Supplementary Data

1.1 RNA isolation and quantitative real-time PCR (qRT-PCR) analysis

TE-1 and Eca-109 cells were treated with 20 µM apigenin or 0.1% DMSO for 48 h and subsequently harvested for quantitative real-time PCR (qRT-PCR) analysis. Total RNA was isolated using the TRIzol reagent according to the manufacturer's instructions. Reverse transcription was performed using a commercial reverse transcription kit (GenStar). The expression levels of hsa-miRNAs and their target genes were quantified by qRT-PCR using SYBR Green PCR Master Mix (GenStar). U6 small nuclear RNA and glyceraldehyde-3-phosphate dehydrogenase (GAPDH) were used as internal controls for miRNA and mRNA normalization, respectively

The mature miRNA sequence of selected miRNAs in table 1 were obtained from miRBase (https://www.mirbase.org/). The Forward primers for miRNA quantification were designed according to the instructions provided with the StarScript III miRNA RT Kit (poly(A) tailing method) (catalog No. A238-10 GenStar) . Briefly, the mature miRNA RNA sequence was converted to a DNA sequence by replacing uracil (U) with thymine (T). The forward primer was then designed based on the full-length mature miRNA sequence to ensure specificity for the mature miRNA rather than its precursor forms. To further improve discrimination against pre-miRNA and other longer RNA species, one adenine (A) residue was added to the 3′ end of the primer, as recommended by the manufacturer. Primer length (18–25 nt), GC content, and melting temperature (~60 ° C) were optimized to ensure compatibility with the universal reverse primer supplied in the kit. The designed forward primer was used together with the Universal miRNA-qPCR-R primer for quantitative PCR analysis provided within the kit. The designed forward primers were listed in Supplementary Table-1.

Supplementary Table 1. qRT-PCR primer sequences used to test miRNA expression.

miRNAs/ genes	Forward 5-Primer sequences-3'
hsa-let-7c-3p	GCGGGTCTTCTTTCTTCTCA
hsa-miR-3177-3p	GCGGCACTGGGGACACGT
hsa-miR-374c-3p	ATAATACAACCTGCTAAGTGCT
hsa-miR-4454	GATCCGAGTCACGGCACCA
hsa-miR-4728	ACCTCCCTCCTGCCCCAG
Has-miR-573	AAGTGATGTGTAACTGATCAGA
hsa-miR-548az-5p	CAAAAGTGATTGTG GTTTTTGC
hsa-miR-33b-5p	GTGCATTGCTGTTGCATTGCA

hsa-miR-4479	GGCCGTGCTCGGAGCAGA
hsa-miR-3198	GAGTCCTGGGGAATGGAGA
hsa-miR-miR-4435	GCCAGAGCTCACACAGAGG
hsa-miR-891a-5p	CAACGAACCTGAGCCACTGA
hsa-miR-675-5p	GGAGAGGGCCCACAGTGA
hsa-miR-3170	CTGGGGTTCTGAGACAGACAGTA
hsa-miR-4421	ACCTGTCTGTGGAAAGGAGCTAA
hsa-miR-153-5p	TTGCATAGTCACAAAAGTGATC

Primers for mRNAs/genes were designed using NCBI Primer-BLAST to span exon–exon junctions and avoid genomic DNA amplification. Amplicon specificity was confirmed by BLAST analysis. The sequence of the designed primers is listed in Supplementary Table 2.

Supplementary Table S2. Primer sequences used for qRT-PCR of mRNA/gene

**Gene**	**Forward primer (5′→3′)**	**Reverse primer (5′→3′)**
ALDH3A2	CCTGGTGCTGACTTTGATGA	AGGGTGATGGTCTTCTTGGT
SEMA3F	AGCTGCTACCTGCTGATGAA	TGGTGGTCTTGATGGTCTTG
MAP4K5	CAGGACTTCAGCATCCTCCT	GCTGGTCTTCTTCAGGTTCC
TRIP13	AGGACTTCCAGGCTGTTTGT	CTGGTGGTCTTCTTCTGGGT
ENPP2	TGTGCTGCTACTTCCTGCTT	AGGGTCTTCTTGGTGGTGAT
CD74	CAGAGCTGCTGCTGACTTAC	TCTGGTGGTCTTCAGGTTGT
MMP15	AGCTGCTGCTACTGCTTTGT	TGGGTCTTCTTGGTGGTGAT
EEF1A2	AGGACTTCAGGCTGGTATGA	CTGGTGGTCTTCTTCAGGGT
FSTL3	CCTGCTACTTCCTGCTGCTT	AGGGTCTTCTTGGTGGTGAT
PIK3IP1	AGCTGCTGACTTCCTGCTAT	TGGTGGTCTTCTTCAGGGTC
CD9	TGGCTTCCTGCTGCTACTTT	AGGGTCTTCTTGGTGGTGAT
RIPOR3	CAGCTTCAGCCTGCTACTTC	TGGGTCTTCTTGGTGGTGAT

AGO2	AGGACTTCAGGCTGCTTTGT	CTGGTGGTCTTCTTCAGGGT
MMP2	CAGGACTTCAGGAGGCTTCT	TGGTGGTCTTCTTCAGGGTC
PDK2	AGCTGCTACTTCCTGCTGCT	AGGGTCTTCTTGGTGGTGAT
SLC39A9	TGGCTGCTACTTCCTGCTAT	TGGGTCTTCTTGGTGGTGAT
TRAM2	AGCTGCTGACTTCCTGCTTT	AGGGTCTTCTTGGTGGTGAT
RFPL3	CAGCTTCAGCCTGCTACTTC	TGGGTCTTCTTGGTGGTGAT
RALBP1	AGGACTTCAGGCTGCTTTGT	CTGGTGGTCTTCTTCAGGGT
COX15	GCTGGAGAAGACCTGAAGAA	TCCAGGTTCTTCTGGTTCTG
RP2	CTGCTGCTGAACAAGAAGAA	GGTGGTCTTCTCCTTGTCCT
MKRN2	TGCTTCAGCCTGAAGTTCAT	AGGGTCTTCTGGTTCTTCTG
SALL4	GCTGGAGAAGACTTCCAGAA	CTGGGTTCTTCCTTCTCCAT
PRKD3	GCTTCCAGTTCCTTCCTCAT	TGGGTCTTCTTCAGGTTCTG
MAPK1	TGGCTGTTCAAGAAGTTCGT	GGCTGTGATGTTCTTGTCCT
NGFR	CAGTTCCTGCTGCTCTTCAT	AGGTCCTTCAGGTTCTCCAG

1.2 Supplementary Figures

Supplementary Figure S1A. Bioinformatics analysis network plots of differentially expressed miRNAs between apigenin-treated and DMSO groups in TE-1 cells.

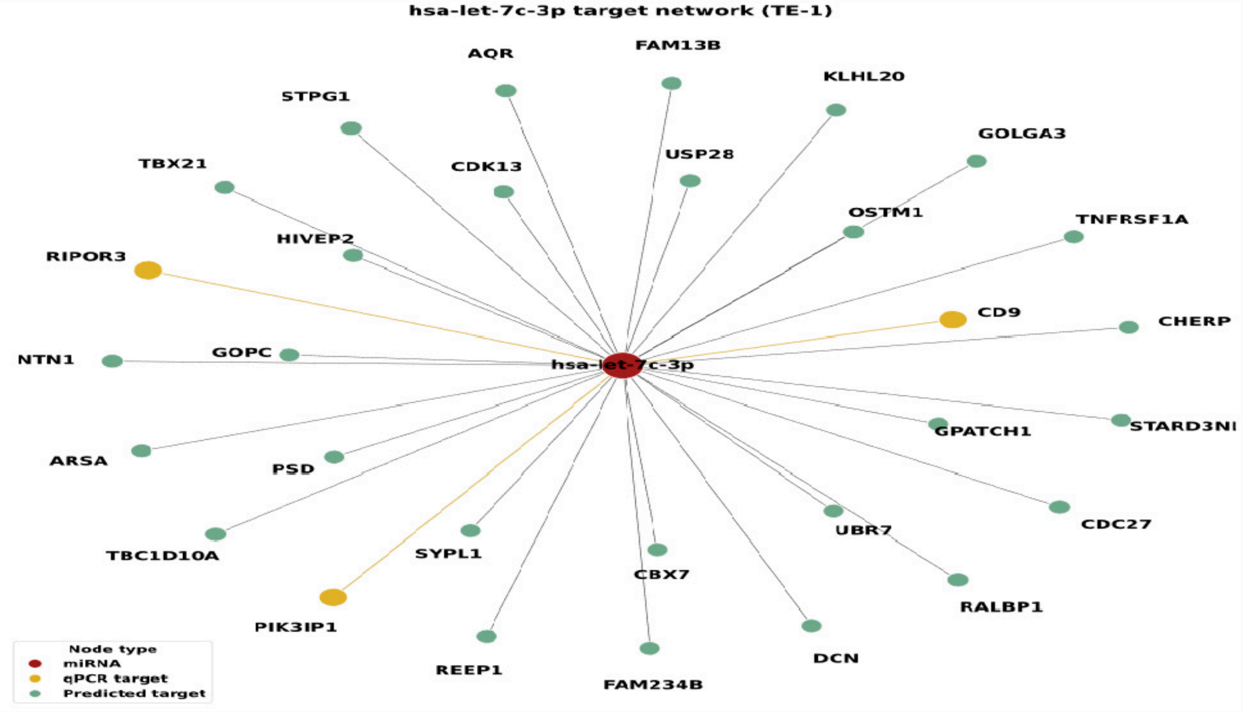
hsa-let-7c-3p target network (TE-1)
AQR
FAM13B
KLHL20
STPG1
GOLGA3
TBX21
CDK13
USP28
OSTM1
TNFRSF1A
HIVEP2
RIPOR3
CD9
CHERP
NTN1
GOPC
hsa-let-7c-3p
ARSA
PSD
GPATCH1
STARD3NL
TBC1D10A
SYPL1
CBX7
UBR7
CDC27
PIK3IP1
RALBP1
REEP1
FAM234B
DCN
Node type
miRNA
qPCR target
Predicted target

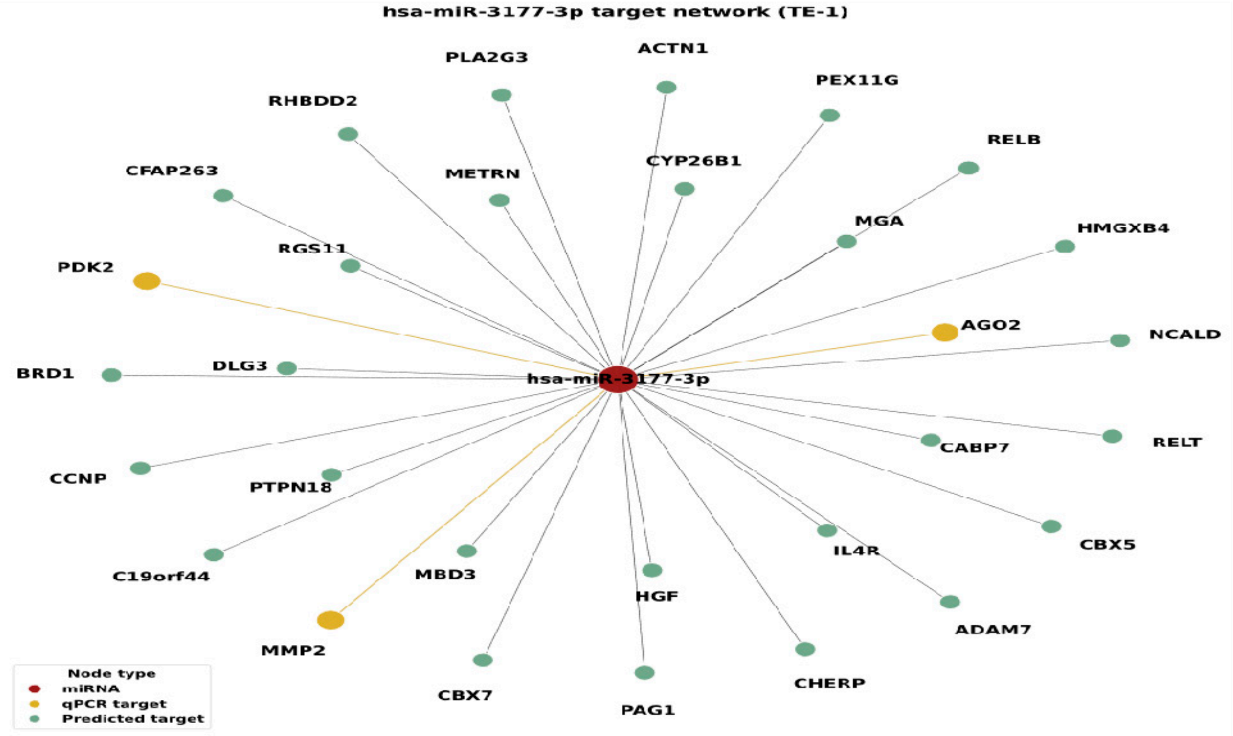
hsa-miR-3177-3p target network (TE-1)
PLA2G3
ACTN1
PEX11G
RHBDD2
RELB
CFAP263
METRN
CYP26B1
MGA
HMGXB4
RGS11
PDK2
AGO2
NCALD
BRD1
DLG3
hsa-miR-3177-3p
RELT
CABP7
CCNP
PTPN18
CBX5
C19orf44
MBD3
IL4R
HGF
ADAM7
MMP2
CBX7
PAG1
CHERP
Node type
miRNA
qPCR target
Predicted target

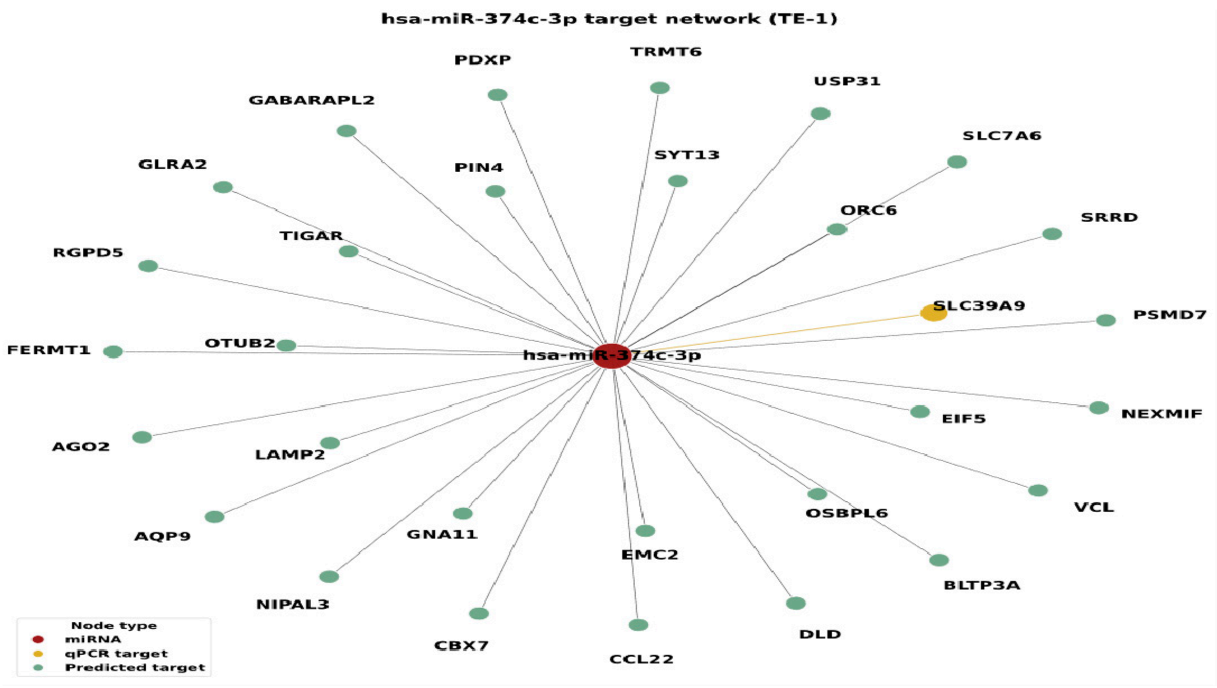
hsa-miR-374c-3p target network (TE-1)
PDXP
TRMT6
USP31
GABARAPL2
SLC7A6
GLRA2
PIN4
SYT13
ORC6
SRRD
TIGAR
RGPD5
SLC39A9
PSMD7
FERMT1
OTUB2
hsa-miR-374c-3p
EIF5
NEXMIF
AGO2
LAMP2
VCL
AQP9
GNA11
OSBPL6
EMC2
BLTP3A
NIPAL3
CBX7
CCL22
DLD
Node type
miRNA
qPCR target
Predicted target

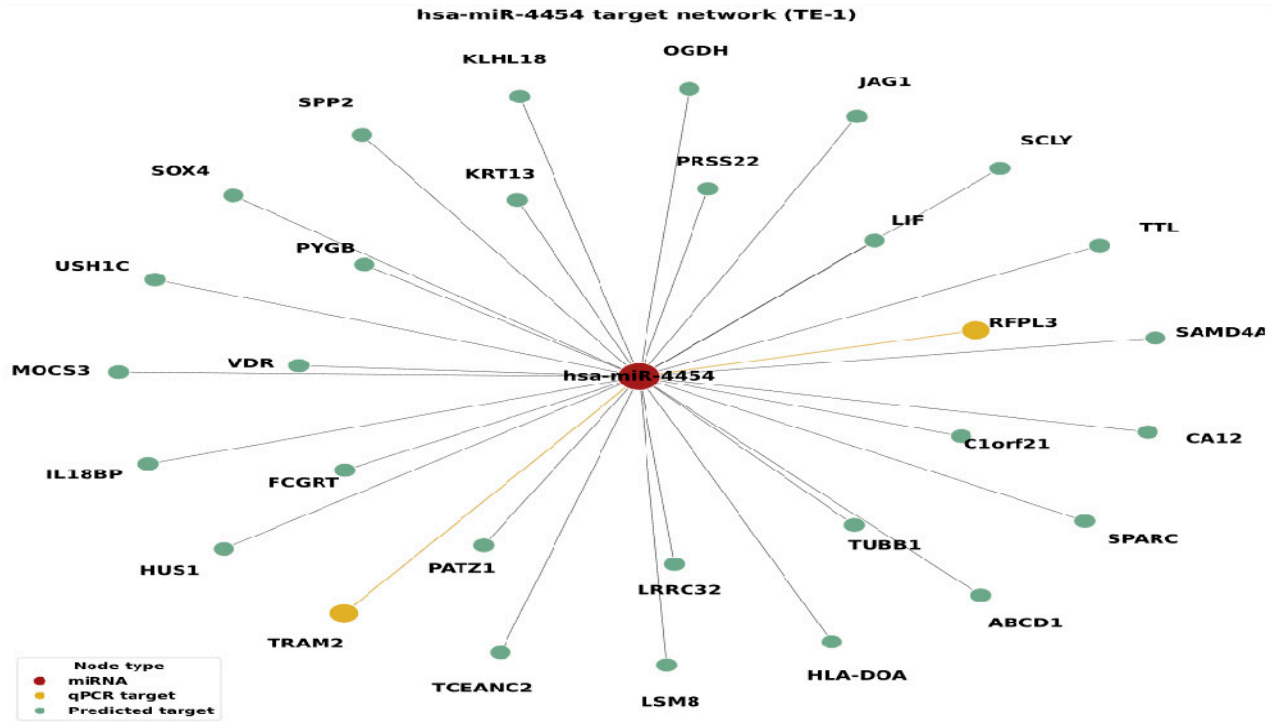
hsa-miR-4454 target network (TE-1)
KLHL18
OGDH
JAG1
SPP2
SCLY
SOX4
KRT13
PRSS22
LIF
TTL
PYGB
USH1C
RFPL3
SAMD4A
MOCS3
VDR
hsa-miR-4454
C1orf21
CA12
IL18BP
FCGRT
SPARC
TUBB1
HUS1
PATZ1
LRRC32
ABCD1
TRAM2
HLA-DOA
TCEANC2
LSM8
Node type
miRNA
qPCR target
Predicted target

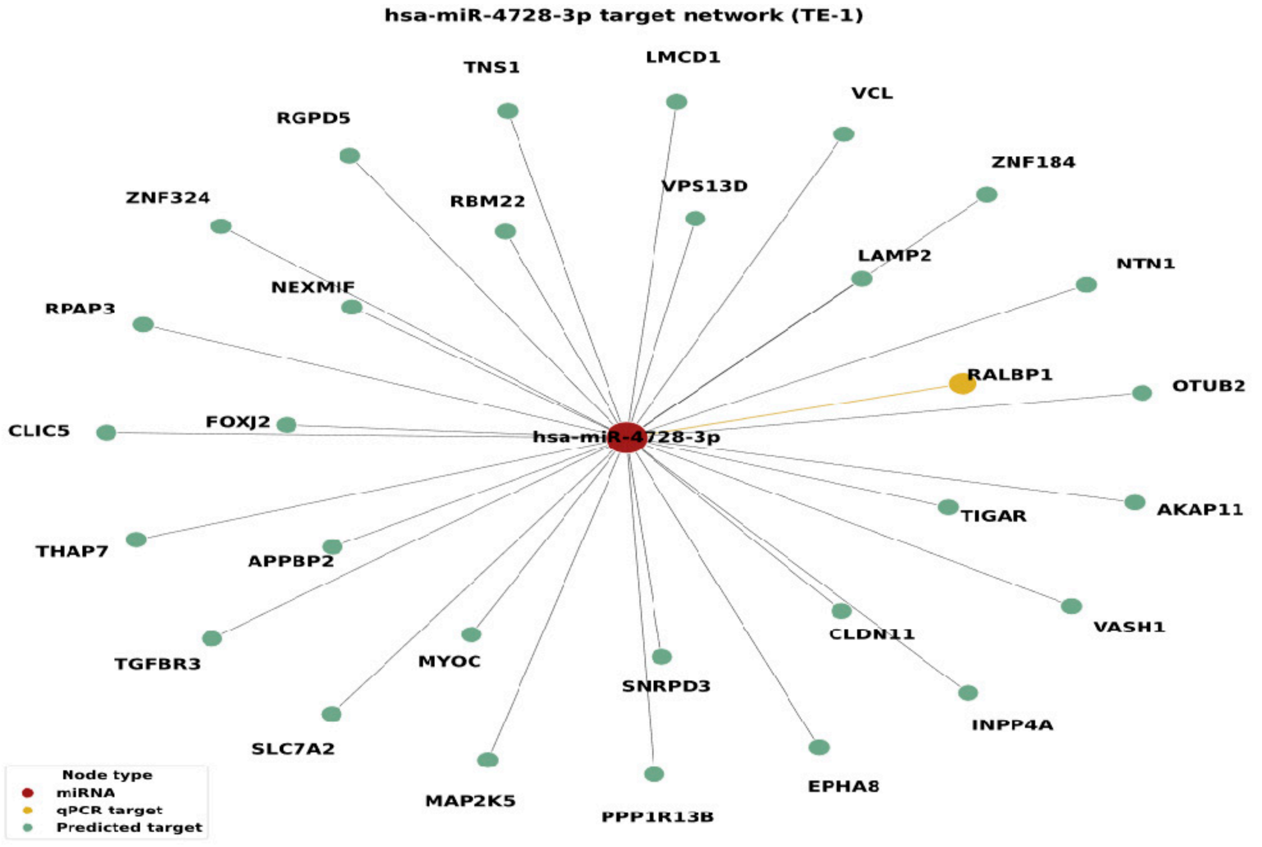

Supplementary Fig. S1A: Upregulated miRNAs following apigenin treatment in TE-1 and their potential validated target genes. hsa-let-7c-3p, hsa-miR-3177-3p, hsa-miR-374c-3p, hsa-miR-4454, and hsa-miR-4728-3p were significantly upregulated upon apigenin treatment. Their corresponding potential target genes were identified through bioinformatic prediction and further validated by quantitative real-time PCR analysis.

Supplementary Figure S1B. Bioinformatics analysis network plots of differentially expressed miRNAs between apigenin-treated and DMSO groups in TE-1 cells.

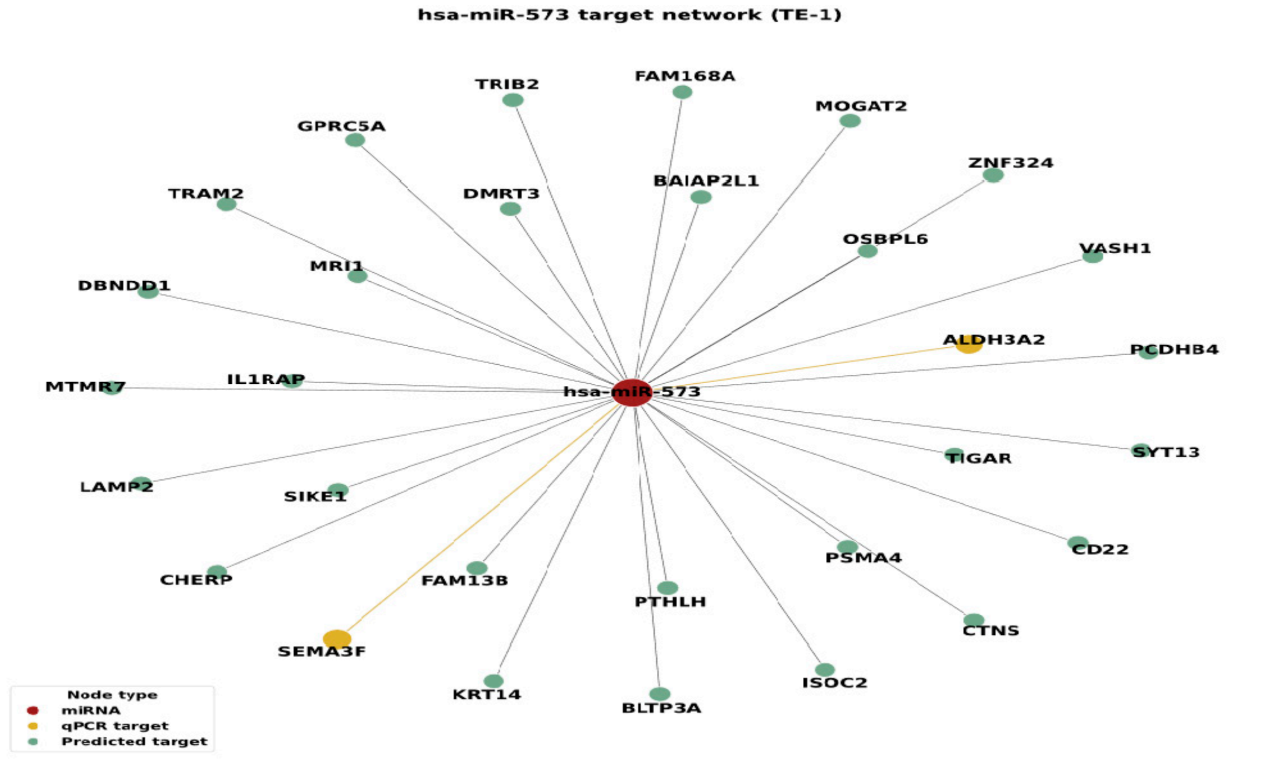

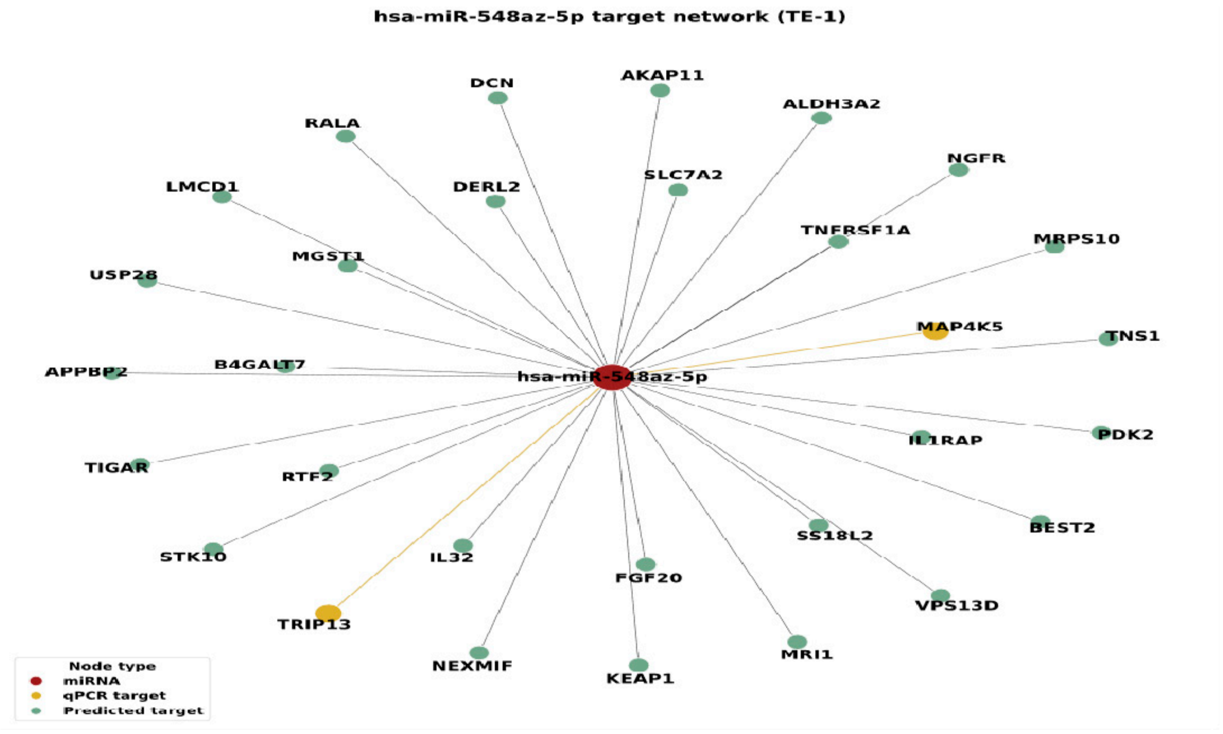

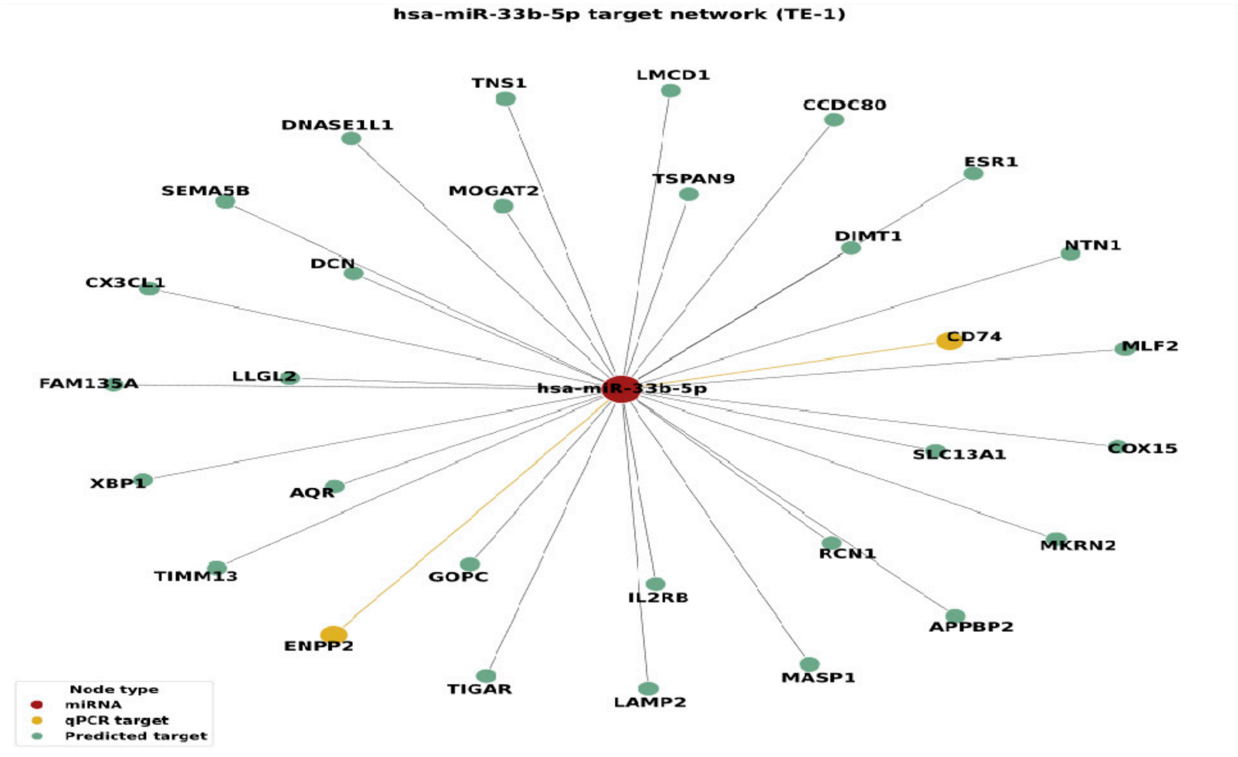
hsa-miR-33b-5p target network (TE-1)
TNS1
LMCD1
CCDC80
DNASE1L1
ESR1
SEMA5B
MOGAT2
TSPAN9
DIMT1
NTN1
DCN
CX3CL1
CD74
MLF2
FAM135A
LLGL2
hsa-miR-33b-5p
XBP1
AQR
SLC13A1
COX15
TIMM13
GOPC
RCN1
MKRN2
IL2RB
ENPP2
APPBP2
TIGAR
LAMP2
MASP1
Node type
miRNA
qPCR target
Predicted target

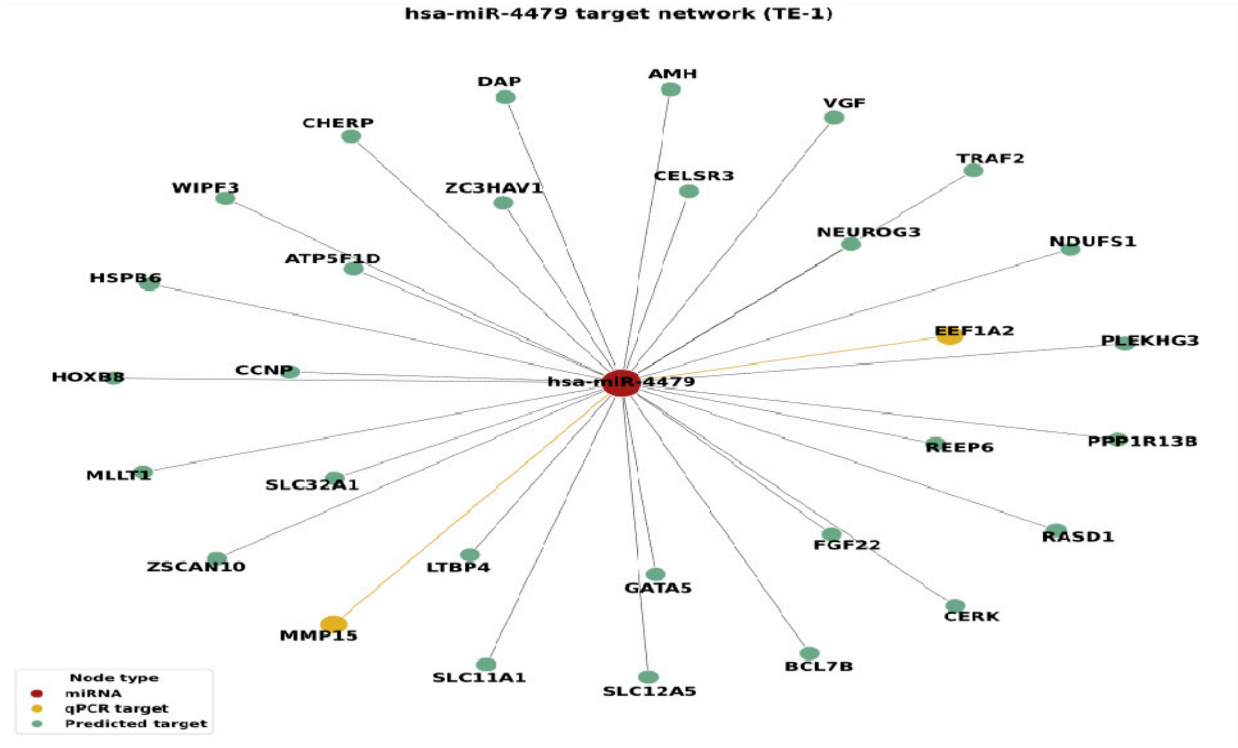
hsa-miR-4479 target network (TE-1)
DAP
AMH
VGF
CHERP
TRAF2
WIPF3
ZC3HAV1
CELSR3
NEUROG3
NDUFS1
ATP5F1D
HSPB6
EEF1A2
PLEKHG3
HOXB8
CCNP
hsa-miR-4479
REEP6
PPP1R13B
MLLT1
SLC32A1
RASD1
ZSCAN10
LTBP4
FGF22
GATA5
CERK
MMP15
SLC11A1
SLC12A5
BCL7B
Node type
miRNA
qPCR target
Predicted target

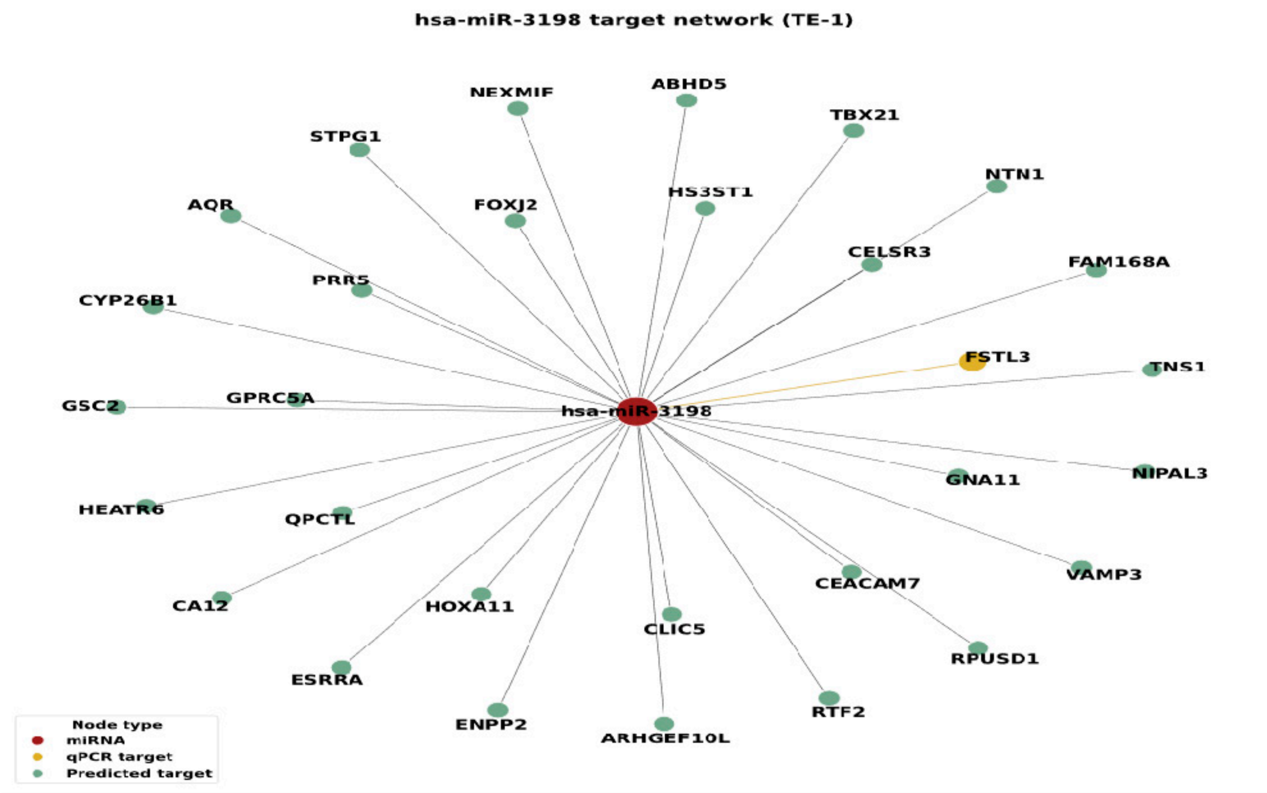

Supplementary Figure S1B. Downregulated miRNAs following apigenin treatment in TE-1 cells and their validated target genes. hsa-miR-573, hsa-miR-548az-5p, hsa-miR-33b-5p, hsa-miR-4479, and hsa-miR-3198 were significantly downregulated upon apigenin treatment. The corresponding target genes were identified through bioinformatic prediction and subsequently validated by quantitative real-time PCR analysis.

Supplementary Figure S2A. Bioinformatics analysis network plots of differentially expressed miRNAs between apigenin-treated and DMSO groups in TE-1 cells.

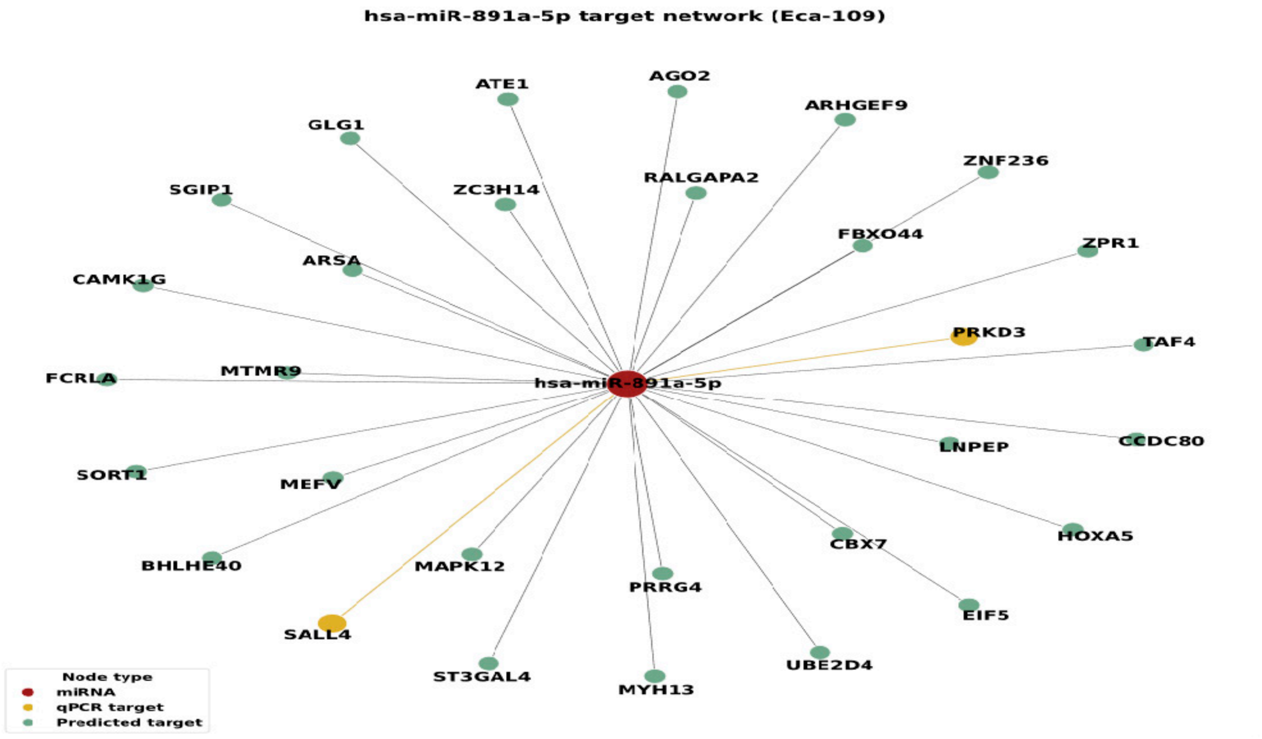

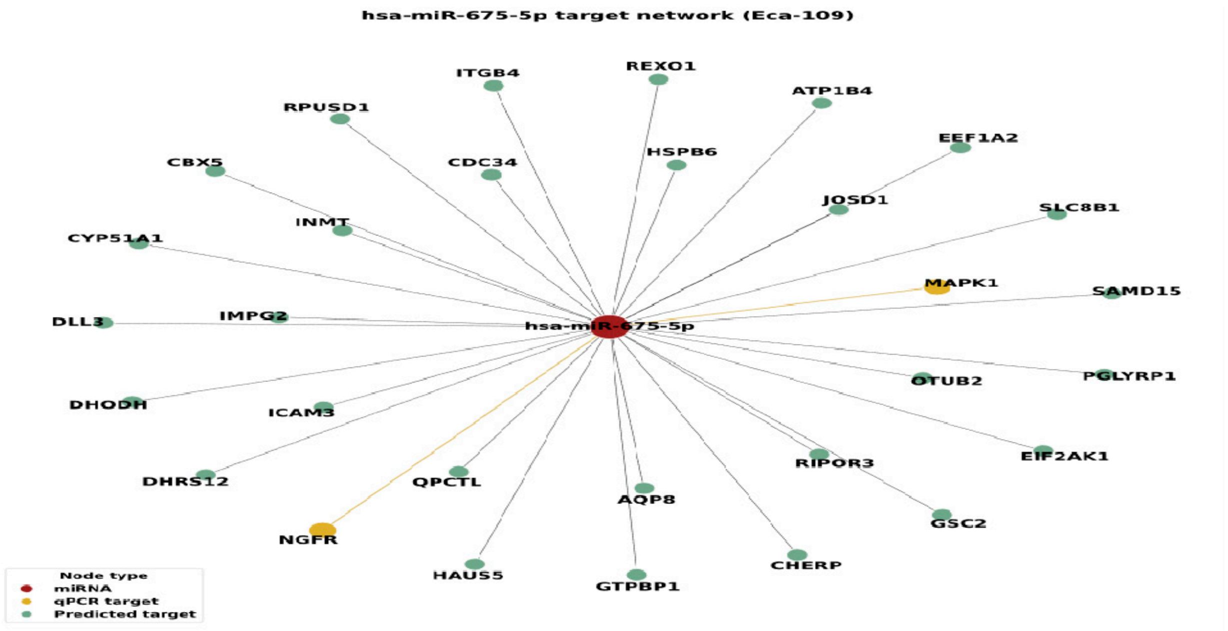

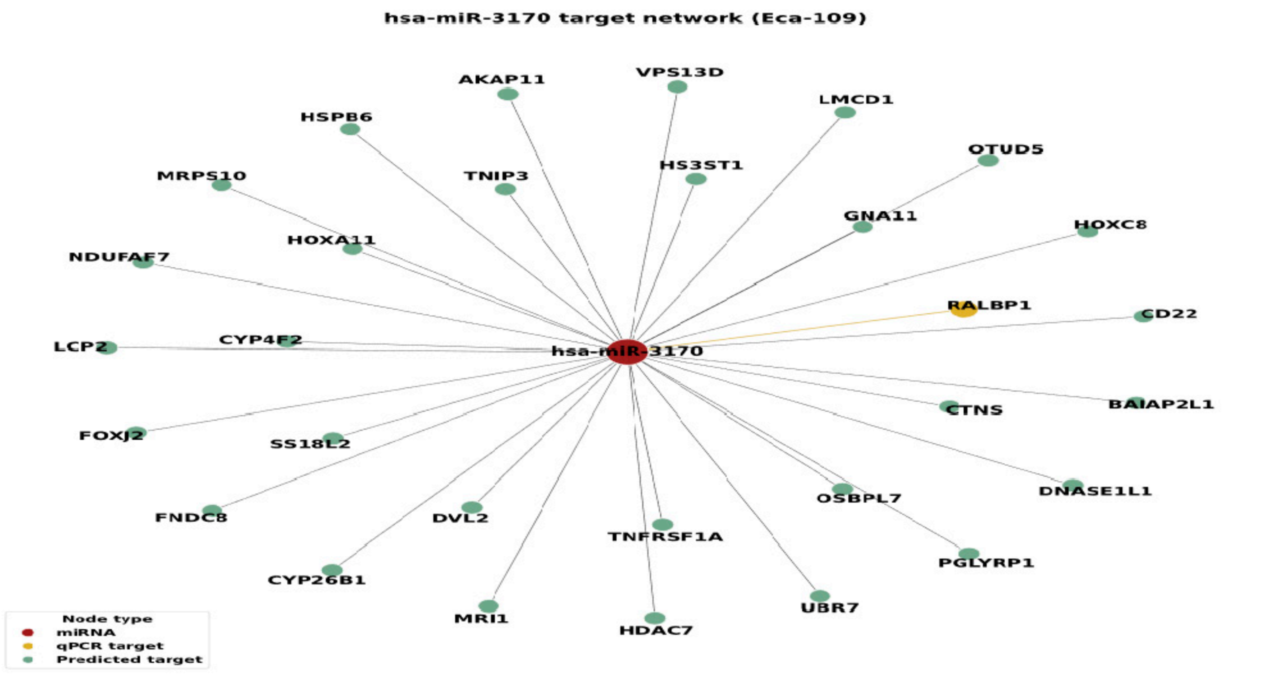
hsa-miR-3170 target network (Eca-109)
hsa-miR-3170
AKAP11
VPS13D
LMCD1
HSPB6
OTUD5
MRPS10
TNIP3
HS3ST1
GNA11
HOXC8
HOXA11
NDUFAF7
RALBP1
CD22
LCP2
CYP4F2
BAIAP2L1
CTNS
FOXJ2
SS18L2
DNASE1L1
OSBPL7
FNDC8
DVL2
TNFRSF1A
PGLYRP1
CYP26B1
UBR7
MRI1
HDAC7
Node type
miRNA
qPCR target
Predicted target

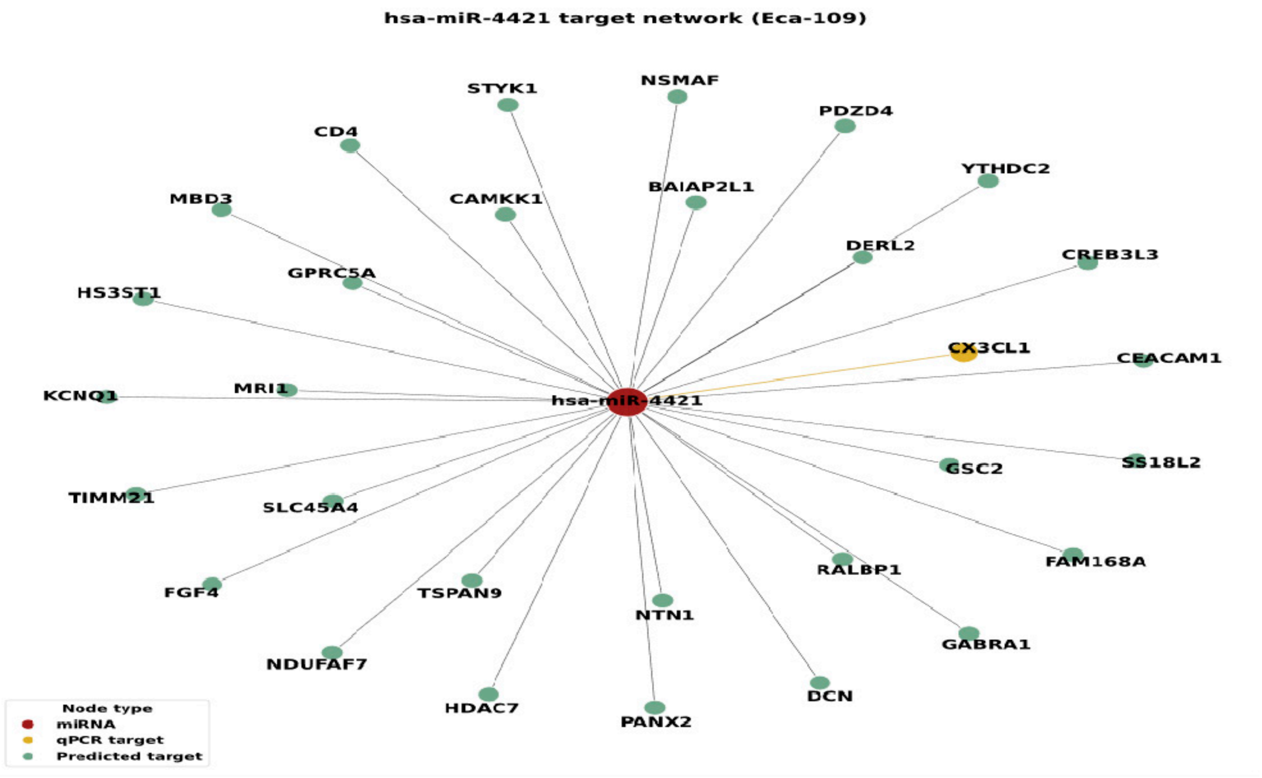
hsa-miR-4421 target network (Eca-109)
hsa-miR-4421
STYK1
NSMAF
PDZD4
CD4
YTHDC2
MBD3
CAMKK1
BAIAP2L1
DERL2
CREB3L3
GPRC5A
HS3ST1
CX3CL1
CEACAM1
KCNQ1
MRI1
SS18L2
GSC2
TIMM21
SLC45A4
FAM168A
RALBP1
FGF4
TSPAN9
NTN1
GABRA1
NDUFAF7
DCN
HDAC7
PANX2
Node type
miRNA
qPCR target
Predicted target

Supplementary Figure S2A. Upregulated miRNAs following apigenin treatment in Eca-109 cells and their validated target genes. hsa-miR-891-5p, hsa-miR-675-5p, hsa-miR-3170, hsa-miR-4421, and hsa-miR-3912 were significantly upregulated upon apigenin treatment. Their corresponding target genes were identified by bioinformatic prediction and subsequently validated by quantitative real-time PCR analysis.

Supplementary Figure S2B. Bioinformatics analysis network plots of differentially expressed miRNAs between apigenin-treated and DMSO groups in Eca-109 cells. miRNA-mRNA networks associated with the up-regulated miRNAs.

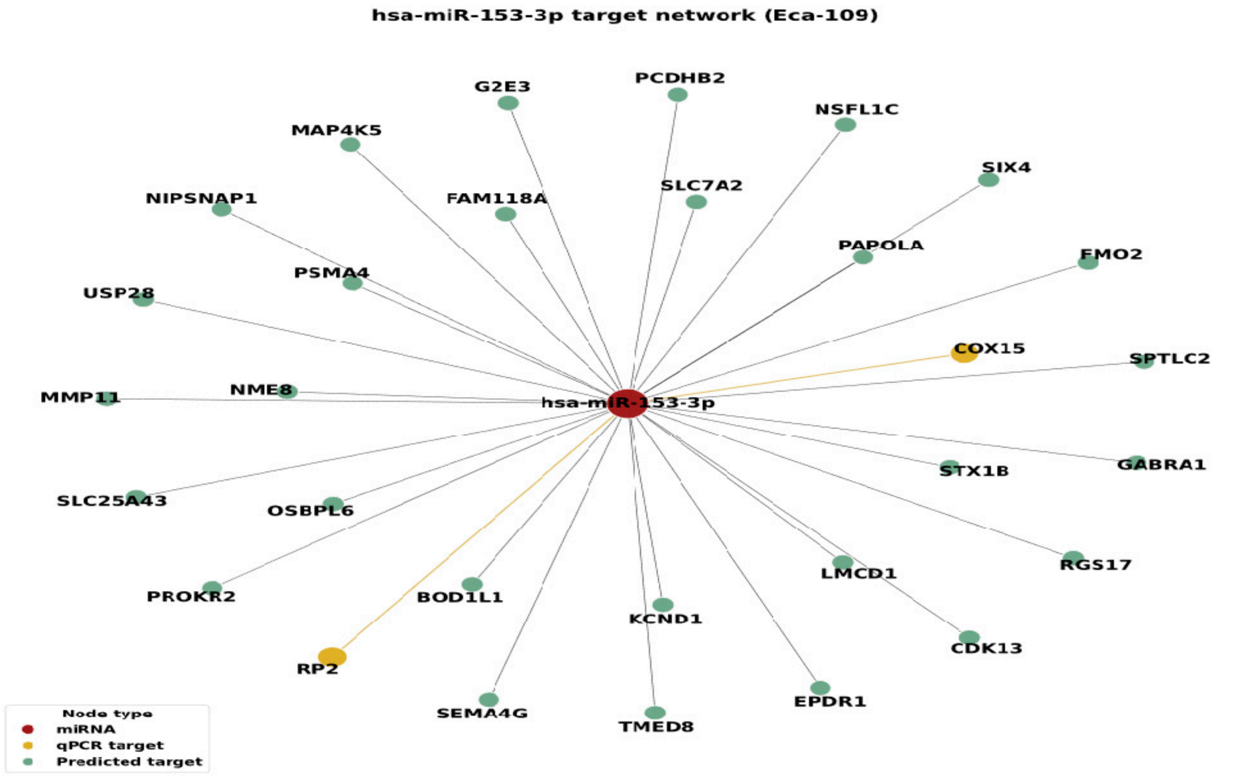

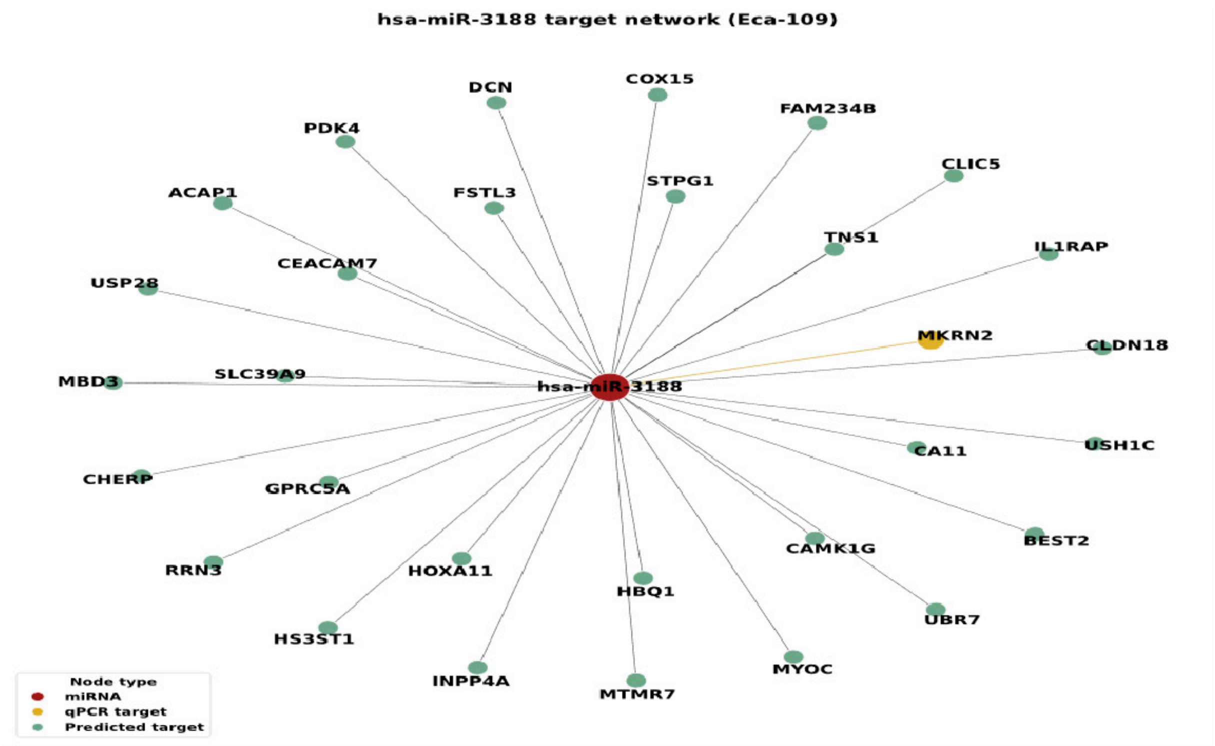
hsa-miR-3188 target network (Eca-109)
DCN
COX15
FAM234B
PDK4
CLIC5
STPG1
FSTL3
ACAP1
TNS1
IL1RAP
CEACAM7
USP28
MKRN2
CLDN18
SLC39A9
MBD3
hsa-miR-3188
USH1C
CA11
CHERP
GPRC5A
BEST2
CAMK1G
RRN3
HOXA11
HBQ1
UBR7
HS3ST1
MYOC
INPP4A
MTMR7
Node type
miRNA
qPCR target
Predicted target

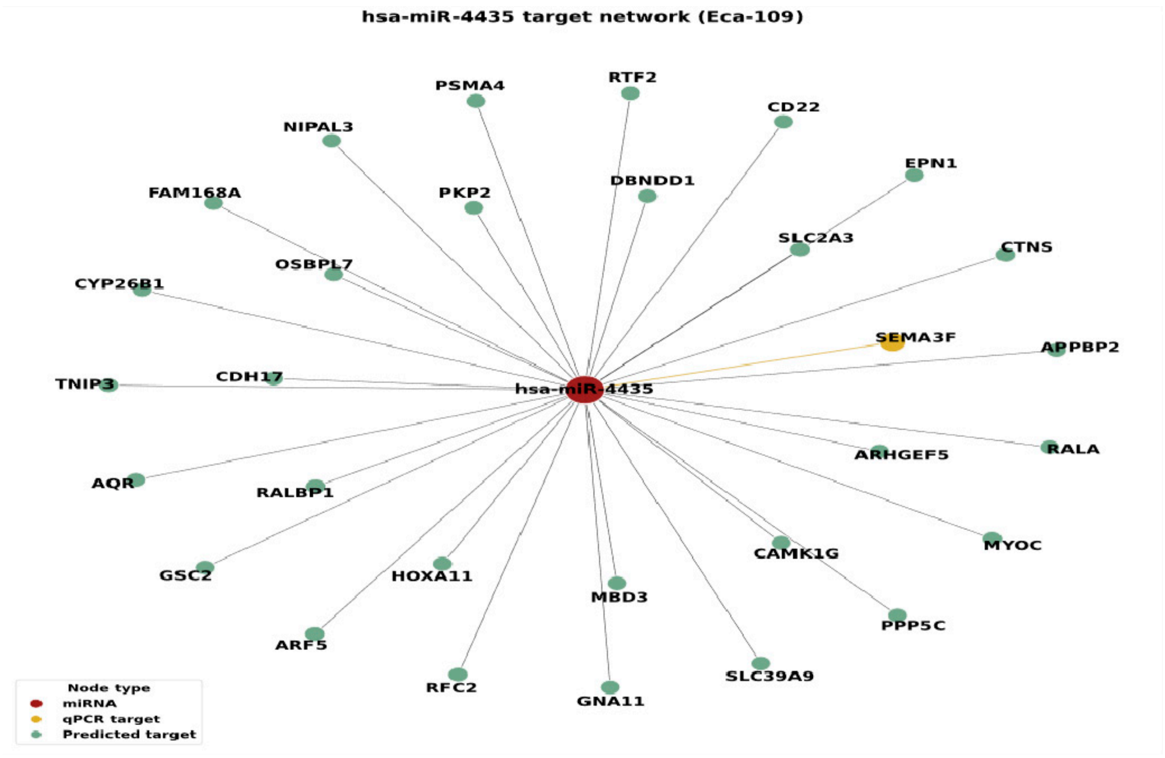
hsa-miR-4435 target network (Eca-109)
RTF2
PSMA4
CD22
NIPAL3
EPN1
FAM168A
PKP2
DBNDD1
SLC2A3
CTNS
OSBPL7
CYP26B1
SEMA3F
APPBP2
CDH17
TNIP3
hsa-miR-4435
RALA
ARHGEF5
AQR
RALBP1
MYOC
CAMK1G
GSC2
HOXA11
MBD3
PPP5C
ARF5
SLC39A9
RFC2
GNA11
Node type
miRNA
qPCR target
Predicted target

Supplementary Figure S2B. Downregulated miRNAs following apigenin treatment in Eca-109 cells and their validated target genes. hsa-miR-153, hsa-miR-3188, hsa-miR-548az-5p, and hsa-miR-4435 were significantly downregulated upon apigenin treatment. The corresponding target genes were identified through bioinformatic prediction and subsequently validated by quantitative real-time PCR analysis.
